# Supplementary material for: Radical Mediated Decarboxylation of Amino Acids via Photochemical Carbonyl Sulfide (COS) Elimination
Source: Molecules. 2024 Mar 25;29(7):1465. doi: 10.3390/molecules29071465 (PMC11013372; doi:10.3390/molecules29071465)
Supplement: Supplementary file 1 [file molecules-29-01465-s001.zip › molecules-2880192-supplementary.pdf]

Supplementary Material
Radical Mediated Decarboxylation of Amino Acids via Photochemical Carbonyl Sulfide (COS) Elimination

Alby Benny [1†], Lorenzo Di Simo [1,2†], Lorenzo Guazzelli [2] and Eoin M. Scanlan[1*]

[1] Trinity Biomedical Sciences Institute, Trinity College Dublin, 152-160 Pearse Street, Dublin 2, D02 R590 Dublin, Ireland
[2] Department of Pharmacy, University of Pisa, Via Bonanno 33, 56126 Pisa, Italy
* Correspondence: eoin.scanlan@tcd.ie
† These authors contributed equally to this work.

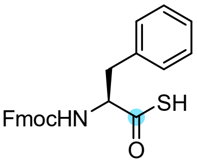

TA-1

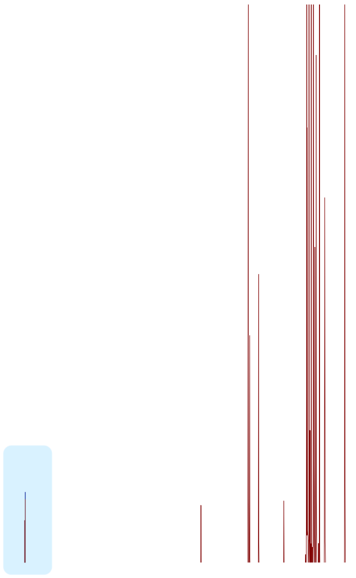

Figure S1. Crude ^{13}C NMR (101 MHz, $CDCl_3$) of thioacid **TA-1** demonstrating complete conversion to the thioacid (δ_C 199.3 ppm) after trityl deprotection. For reference, the thioester carbonyl of Fmoc-Phe-STrt **1a** is found at δ_C 197.2 ppm [11].

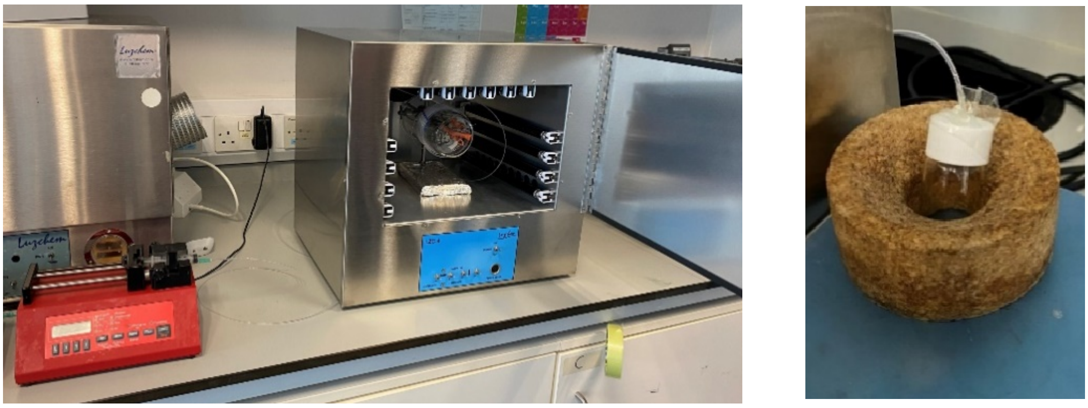

Figure S2. UV flow reactor set-up. Syringe pump with a 10 mL syringe containing the reaction mixture was connected to FEP tubing (inner diameter 0.8 mm) coiled around a glass insert placed inside a Luzchem photoreactor, LZC-EDU (110 V/60 Hz) containing 14 UVA lamps centred at 354 nm (left). The terminus of the tubing was inserted into a glass vial outside the UV reactor for sample collection (right).

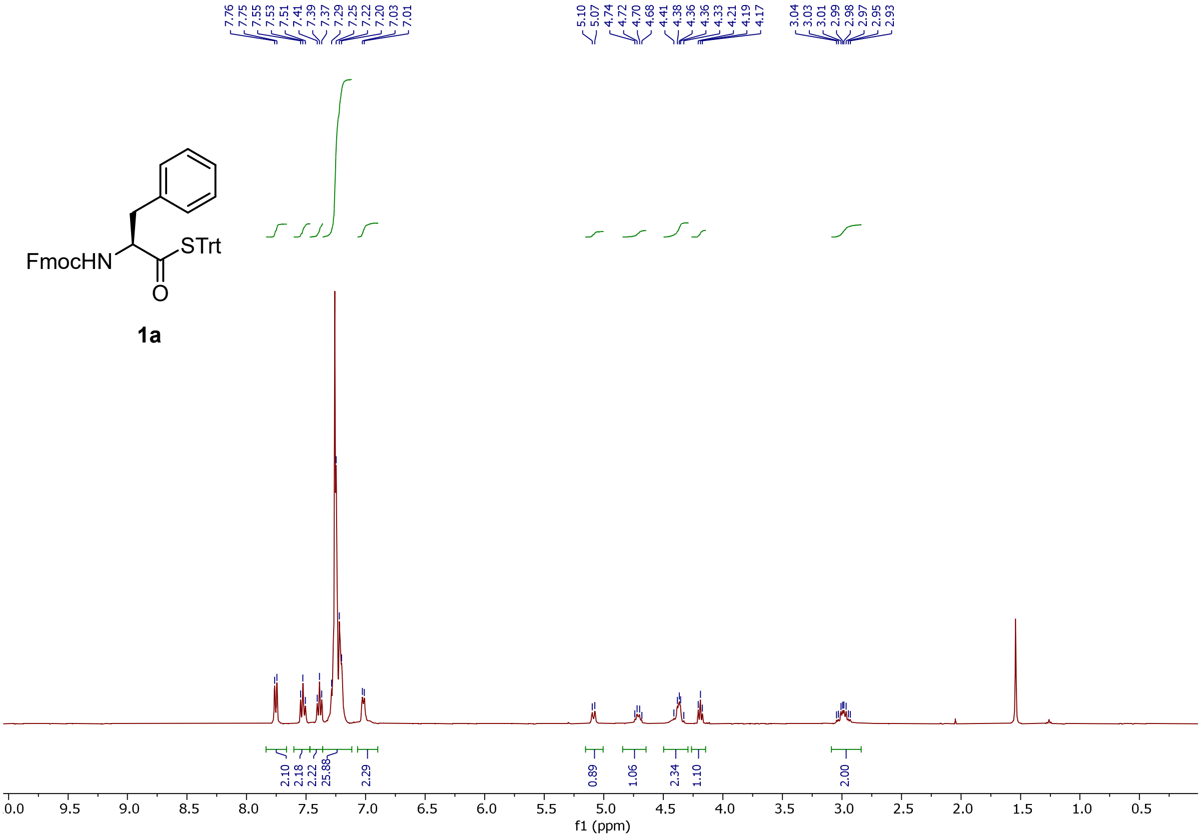

Figure S3. ^{1}H NMR (400 MHz, $CDCl_3$) of compound **1a**.

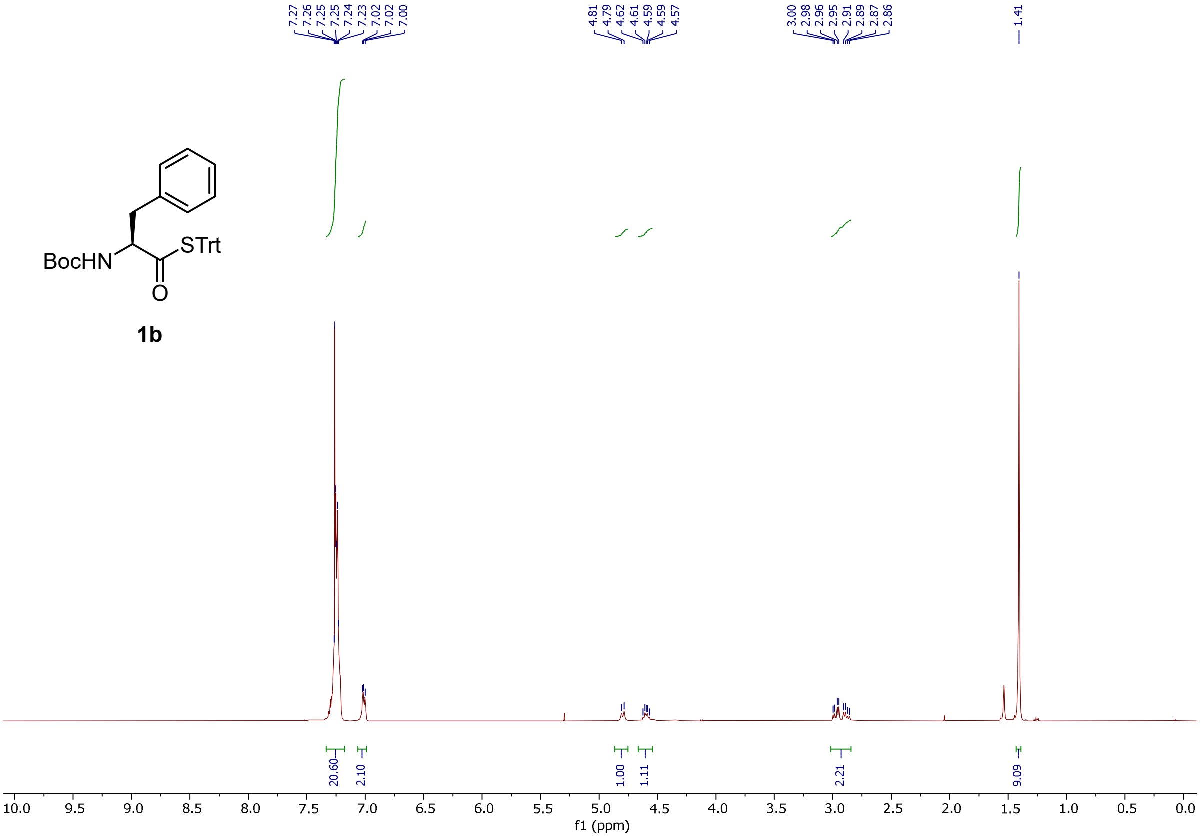

Figure S4. ^{1}H NMR (400 MHz, $CDCl_3$) of compound **1b**.

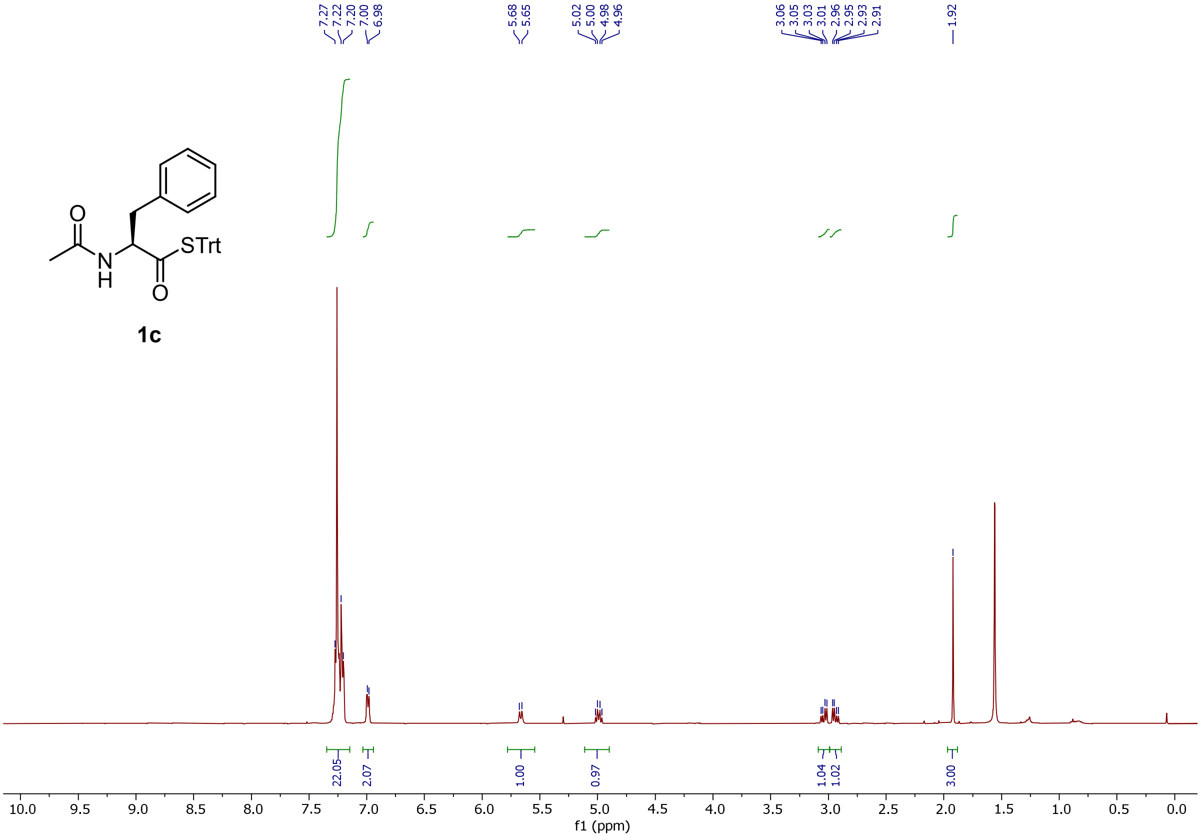

Figure S5. ^{1}H NMR (400 MHz, $CDCl_3$) of compound **1c**.

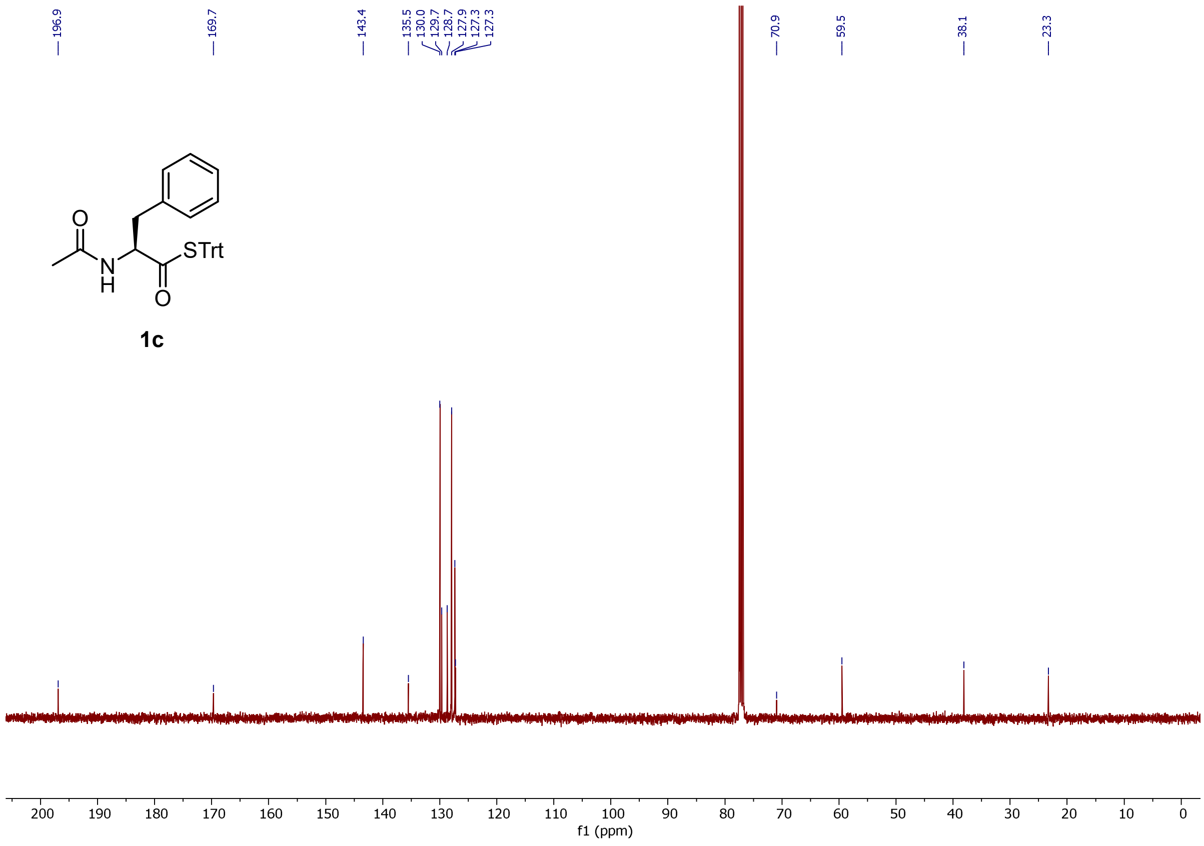

Figure S6. ^{13}C NMR (101 MHz, $CDCl_3$) of compound **1c**.

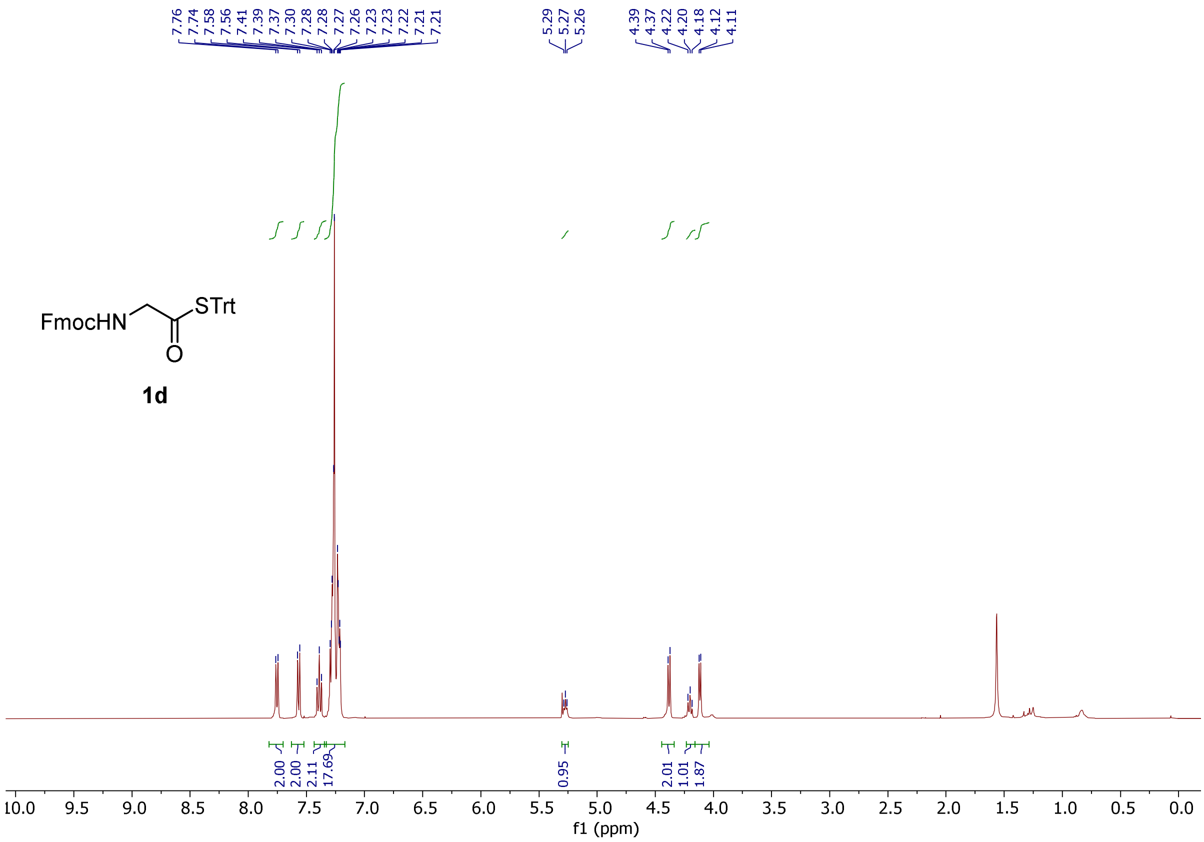

Figure S7. ^{1}H NMR (400 MHz, $CDCl_3$) of compound **1d**.

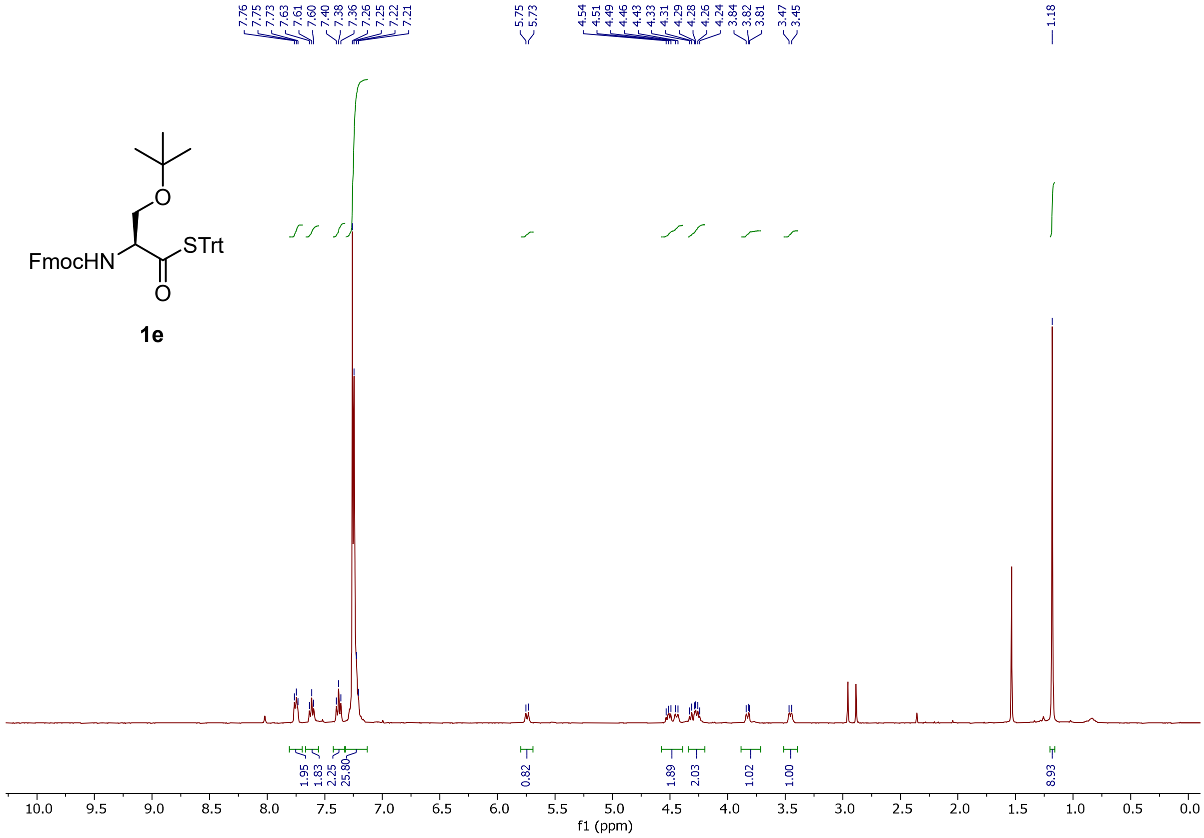

Figure S8. ^{1}H NMR (400 MHz, $CDCl_3$) of compound **1e**.

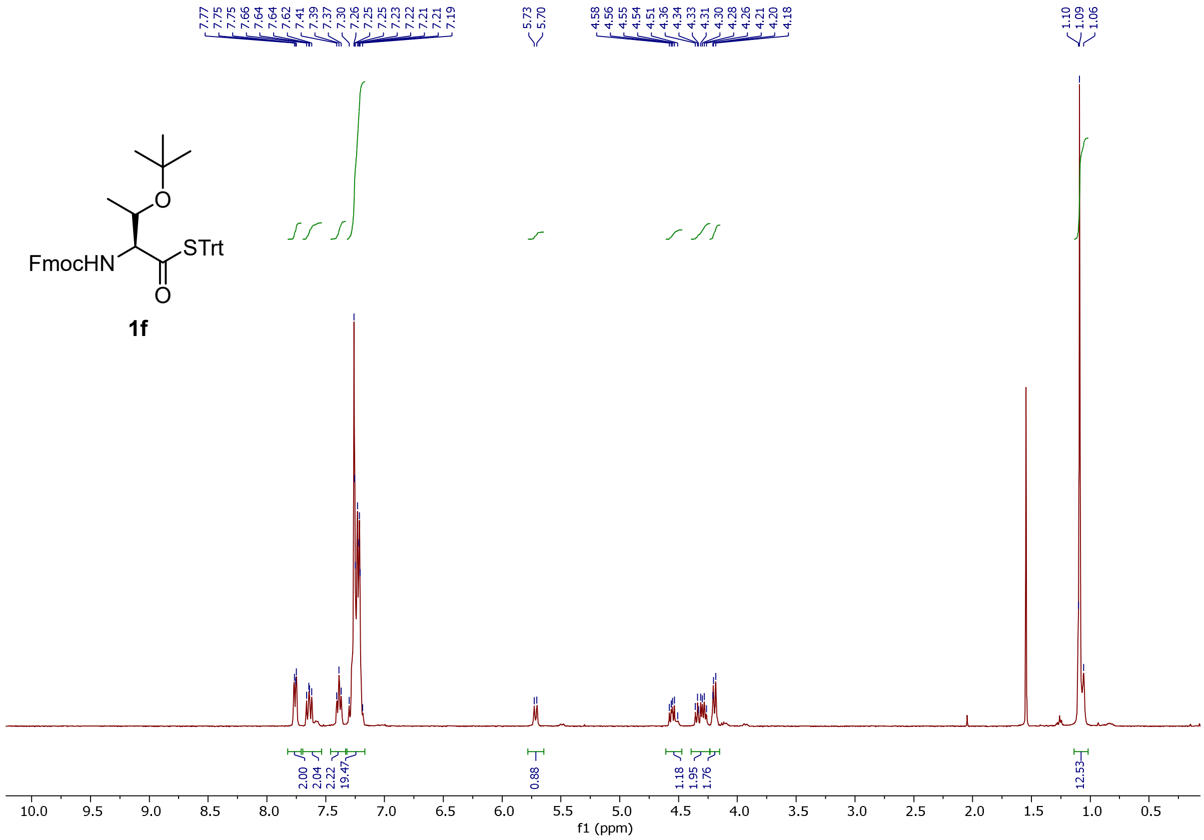

Figure S9. ^{1}H NMR (400 MHz, $CDCl_3$) of compound **1f**.

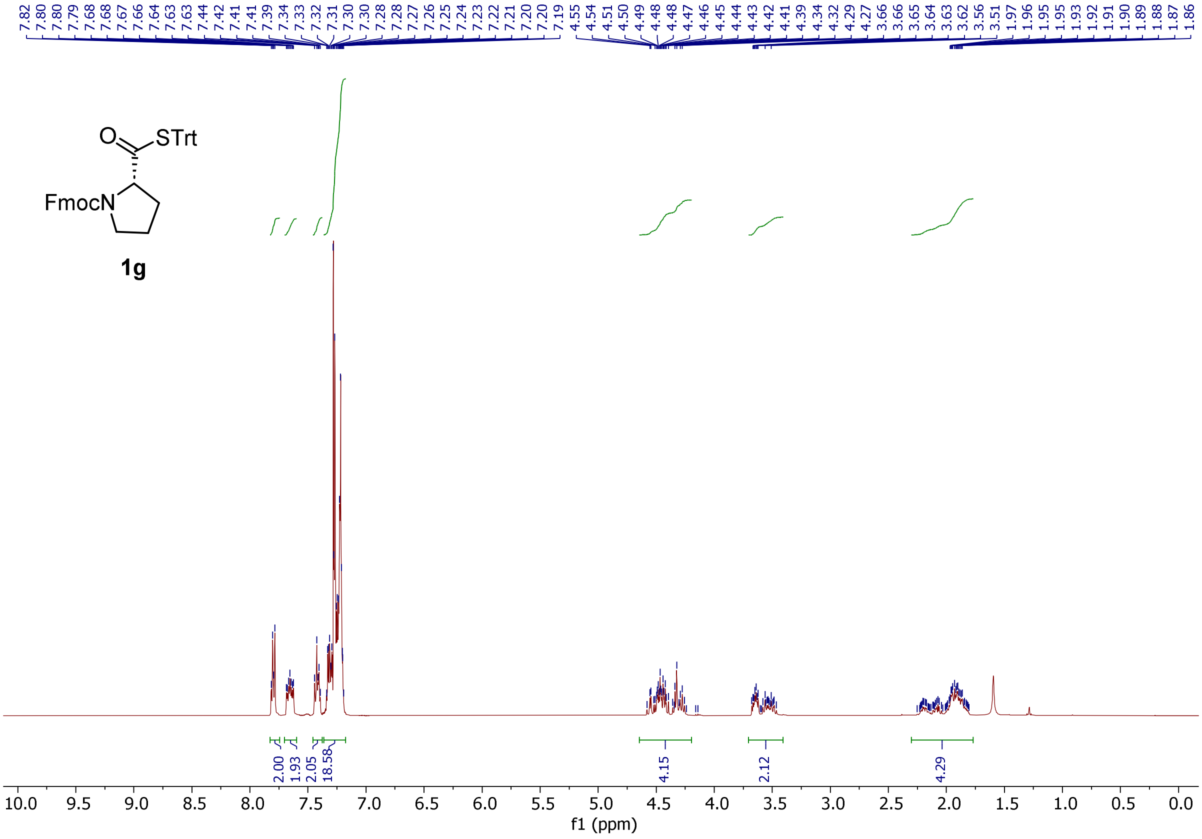

Figure S10. ^{1}H NMR (400 MHz, $CDCl_3$) of compound **1g**.

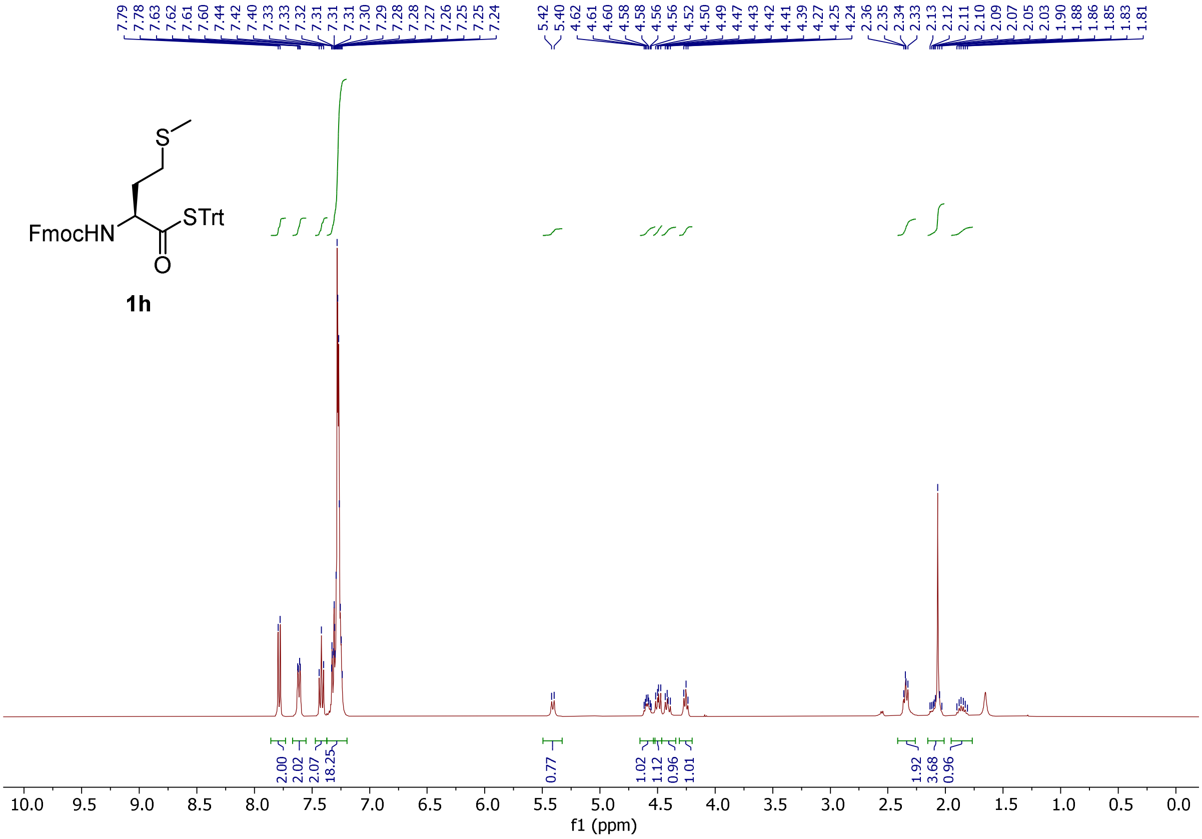

Figure S11. ^{1}H NMR (400 MHz, $CDCl_3$) of compound **1h**.

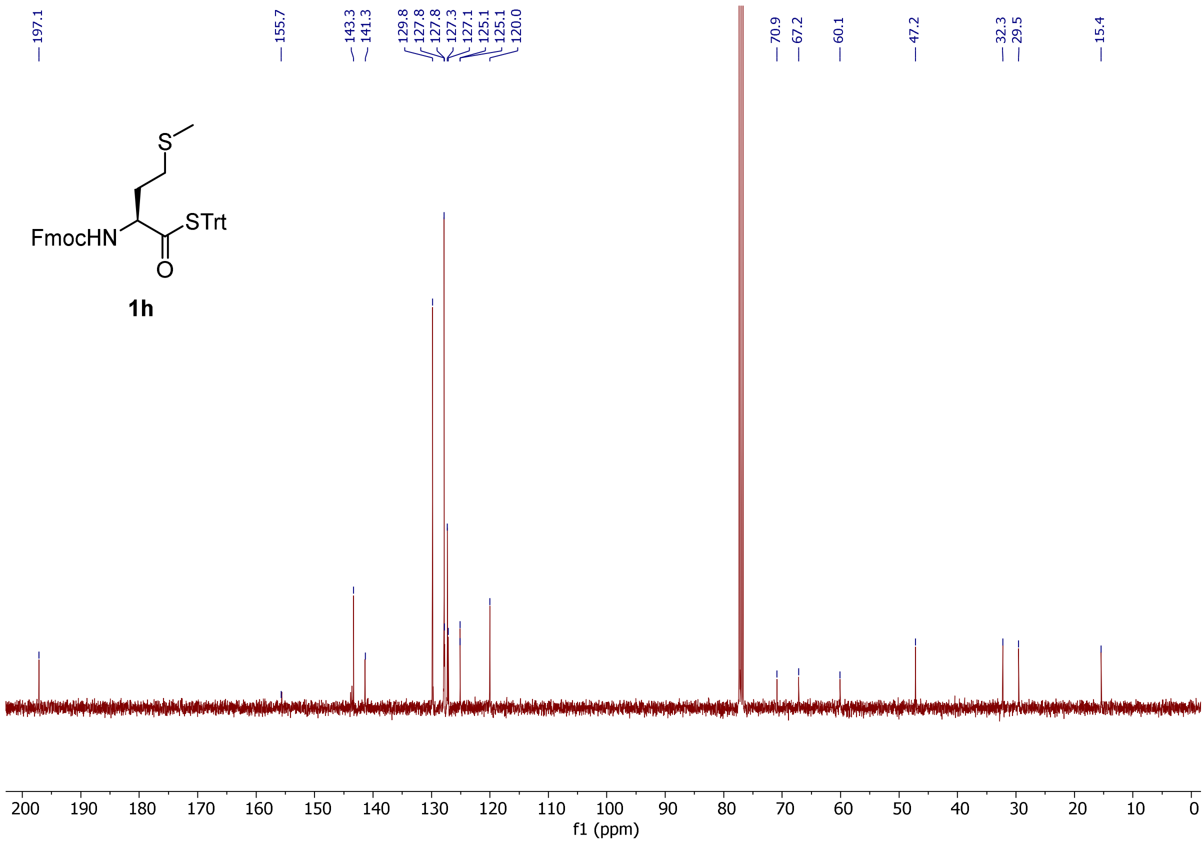

Figure S12. ^{13}C NMR (101 MHz, $CDCl_3$) of compound **1h**.

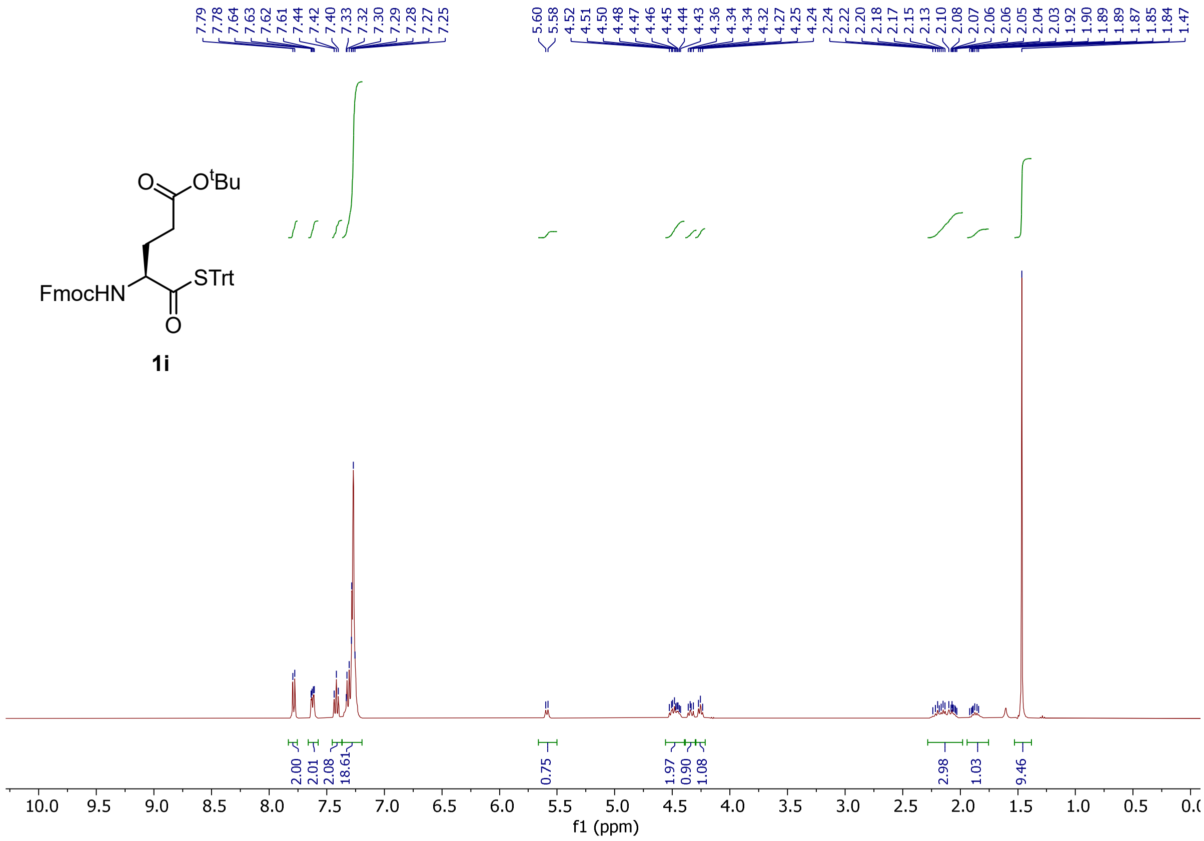

Figure S13. ^{1}H NMR (400 MHz, $CDCl_3$) of compound **1i**.

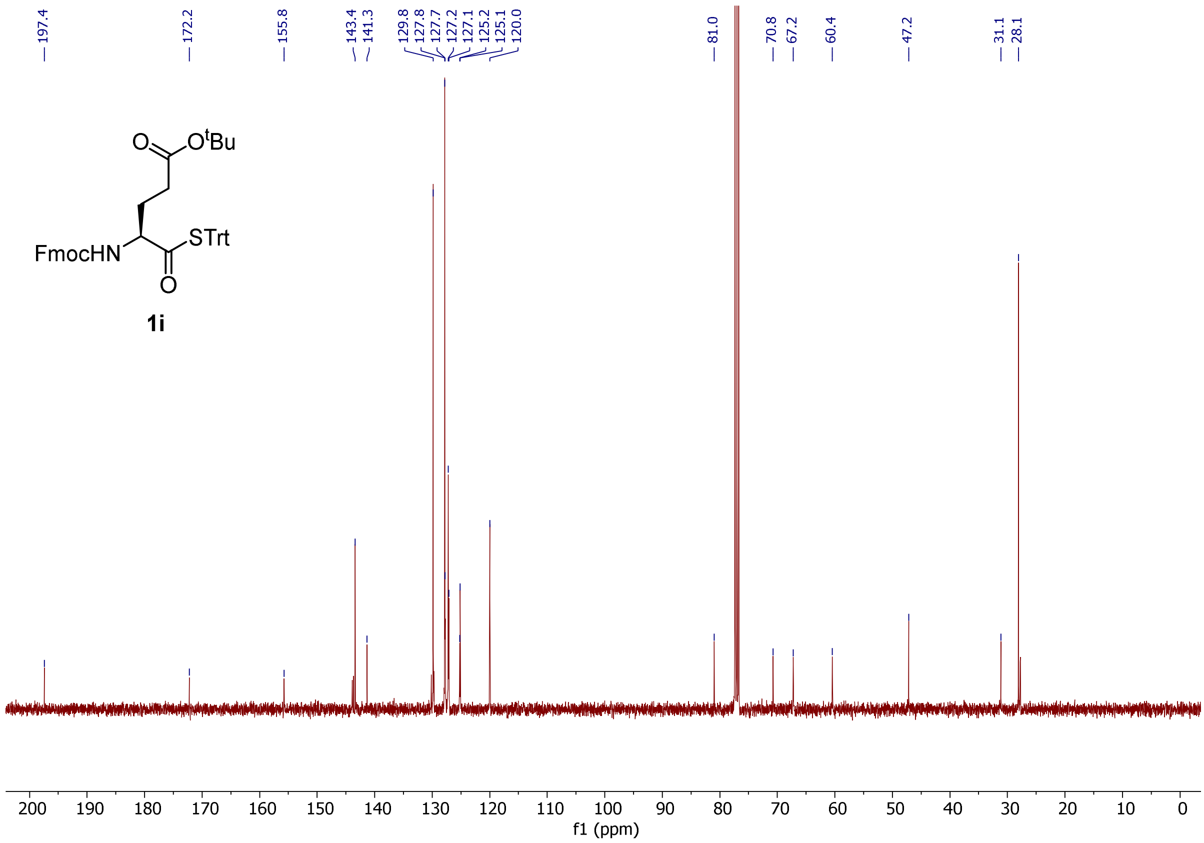

Figure S14. ^{13}C NMR (101 MHz, CDCl$_3$) of compound **1i**.

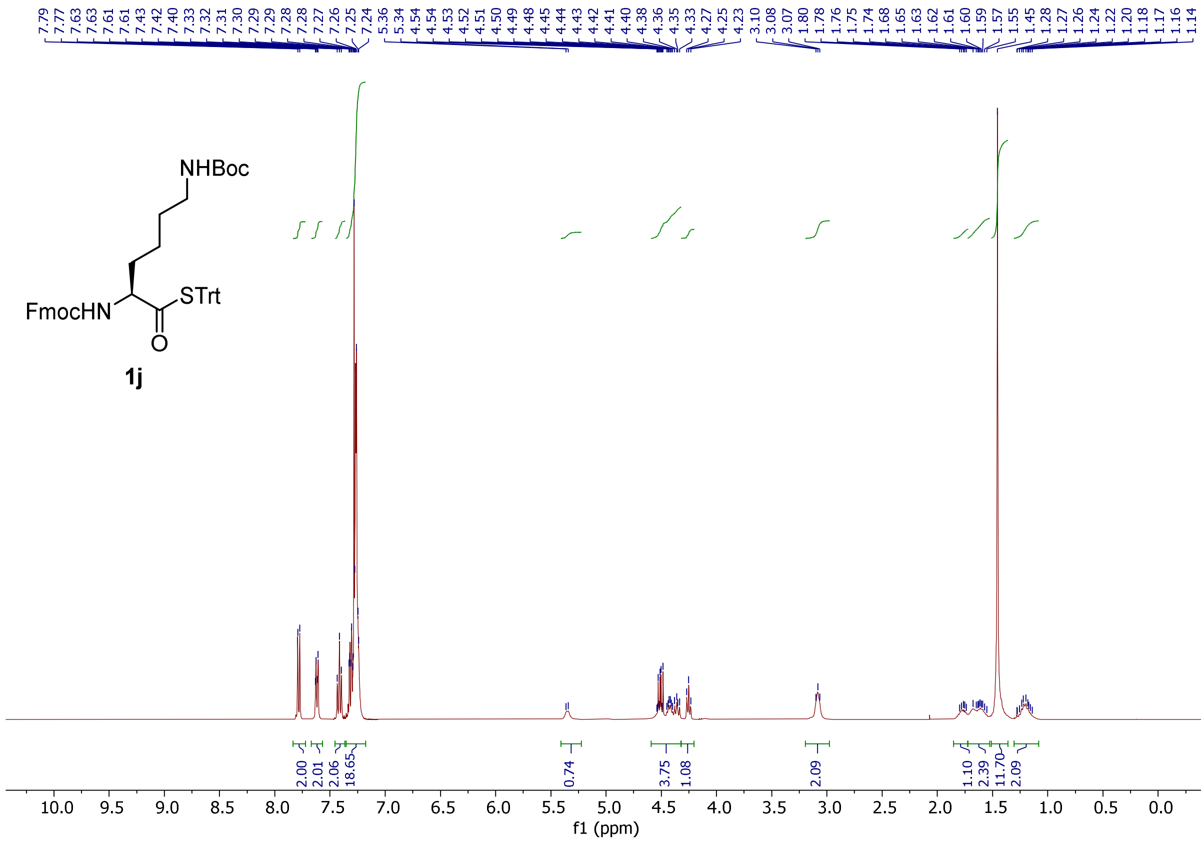

Figure S15. ^{1}H NMR (400 MHz, CDCl$_3$) of compound **1j**.

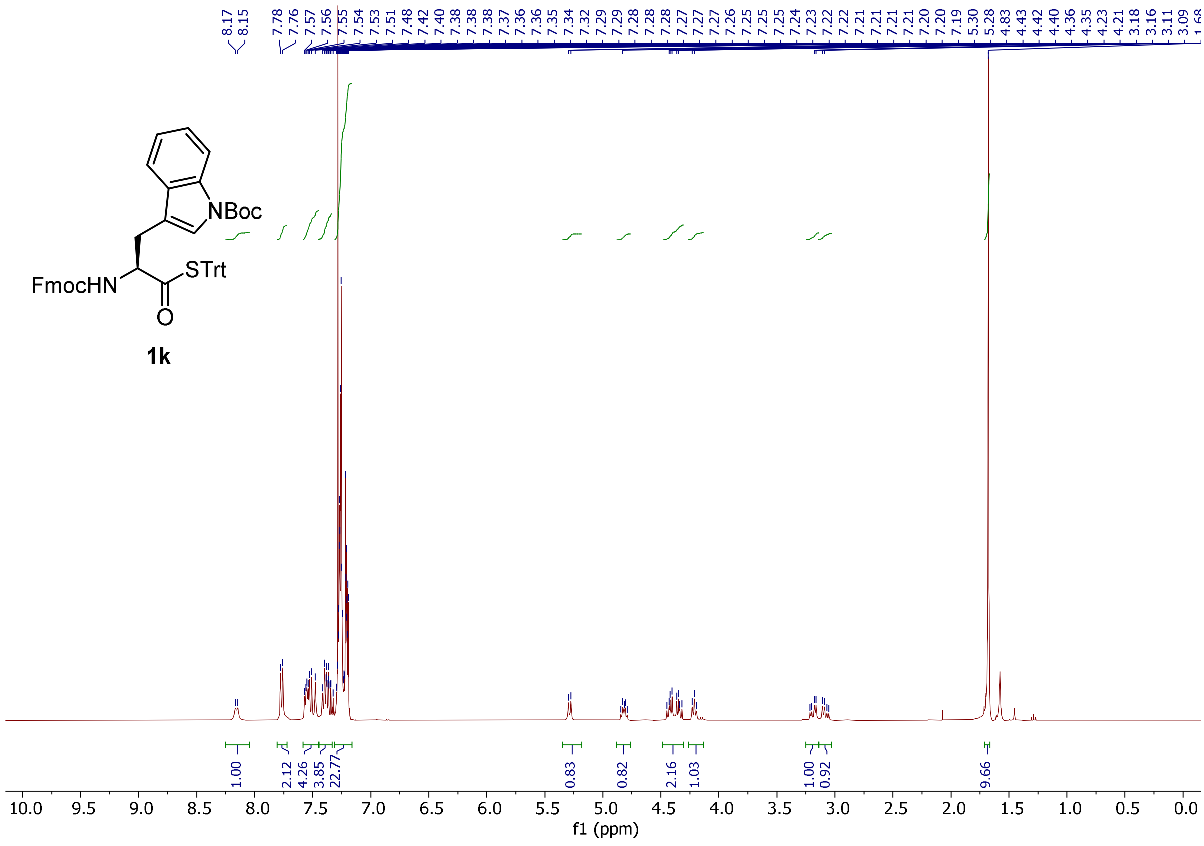

Figure S16. ^{1}H NMR (400 MHz, $CDCl_3$) of compound **1k**.

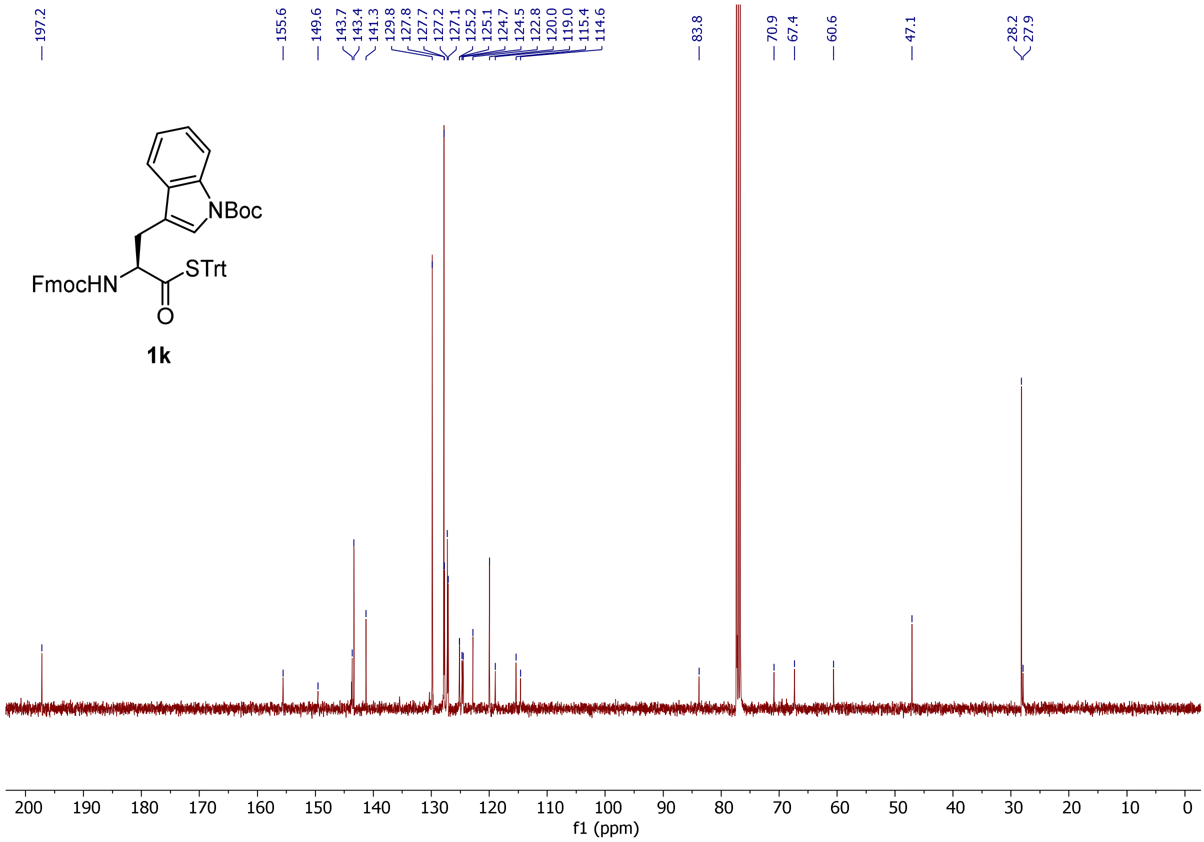

Figure S17. ^{13}C NMR (101 MHz, $CDCl_3$) of compound **1k**.

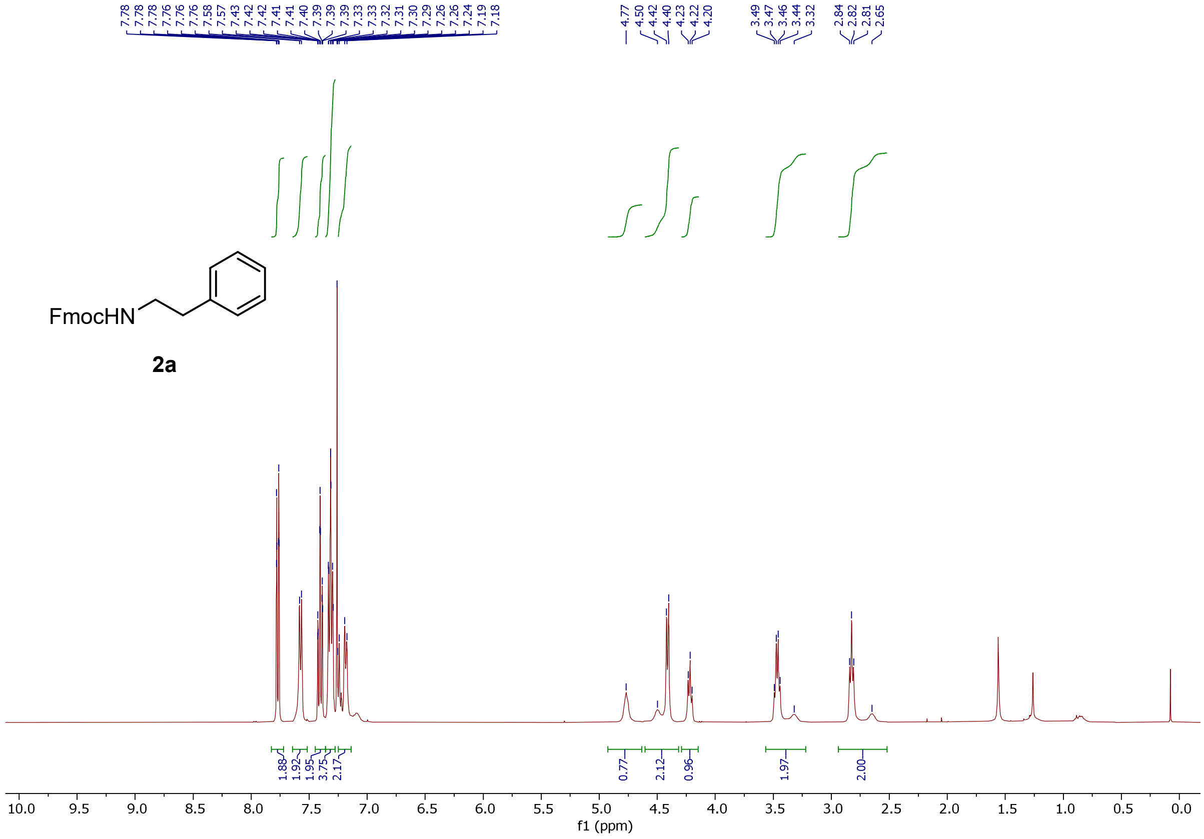

Figure S18. ^{1}H NMR (400 MHz, $CDCl_3$) of compound **2a**.

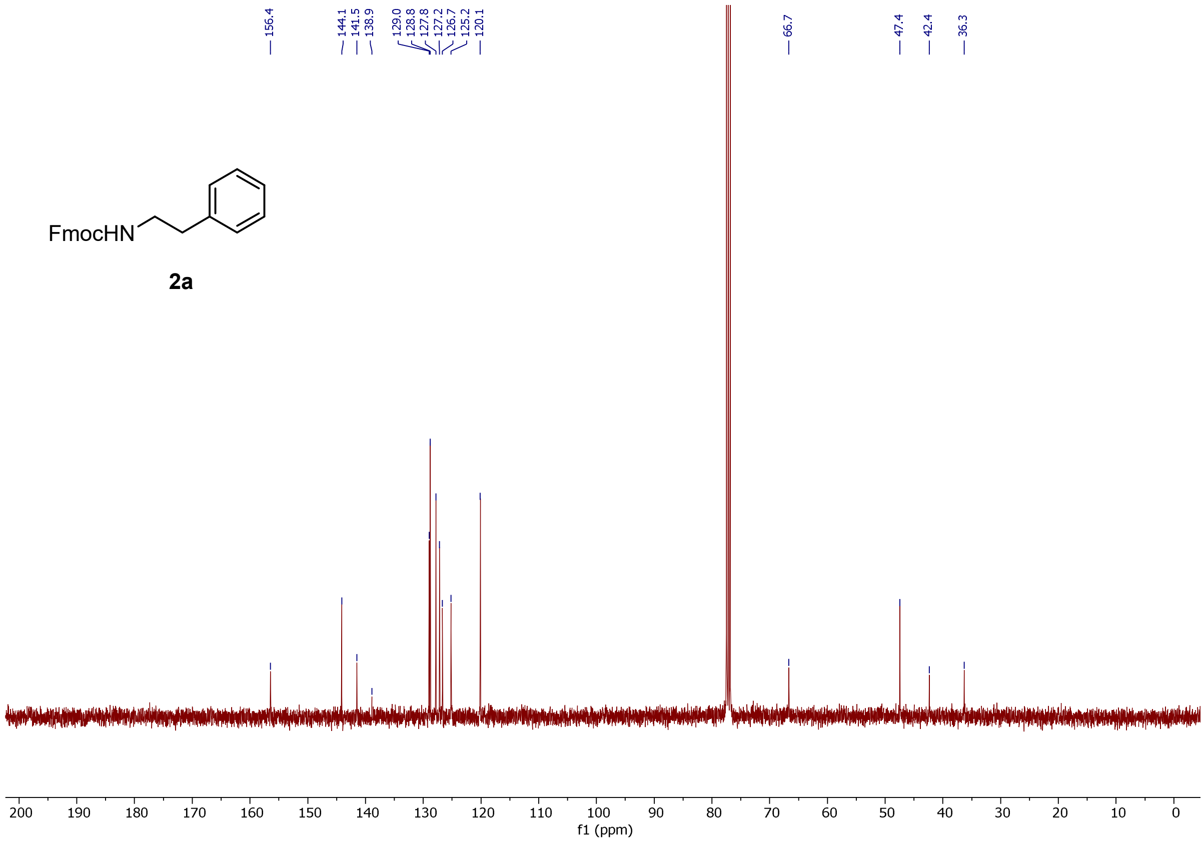

Figure S19. ^{13}C NMR (101 MHz, $CDCl_3$) of compound **2a**.

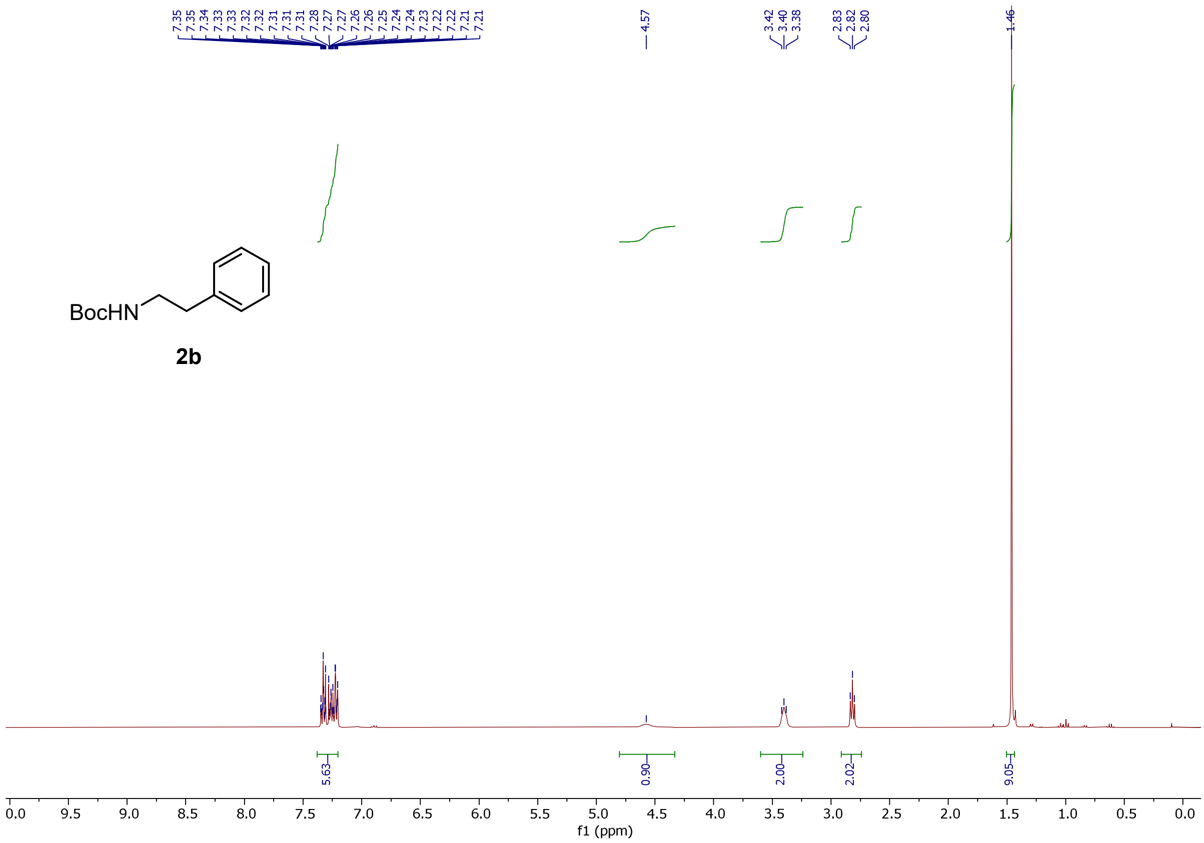

Figure S20. ^{1}H NMR (400 MHz, $CDCl_3$) of compound **2b**.

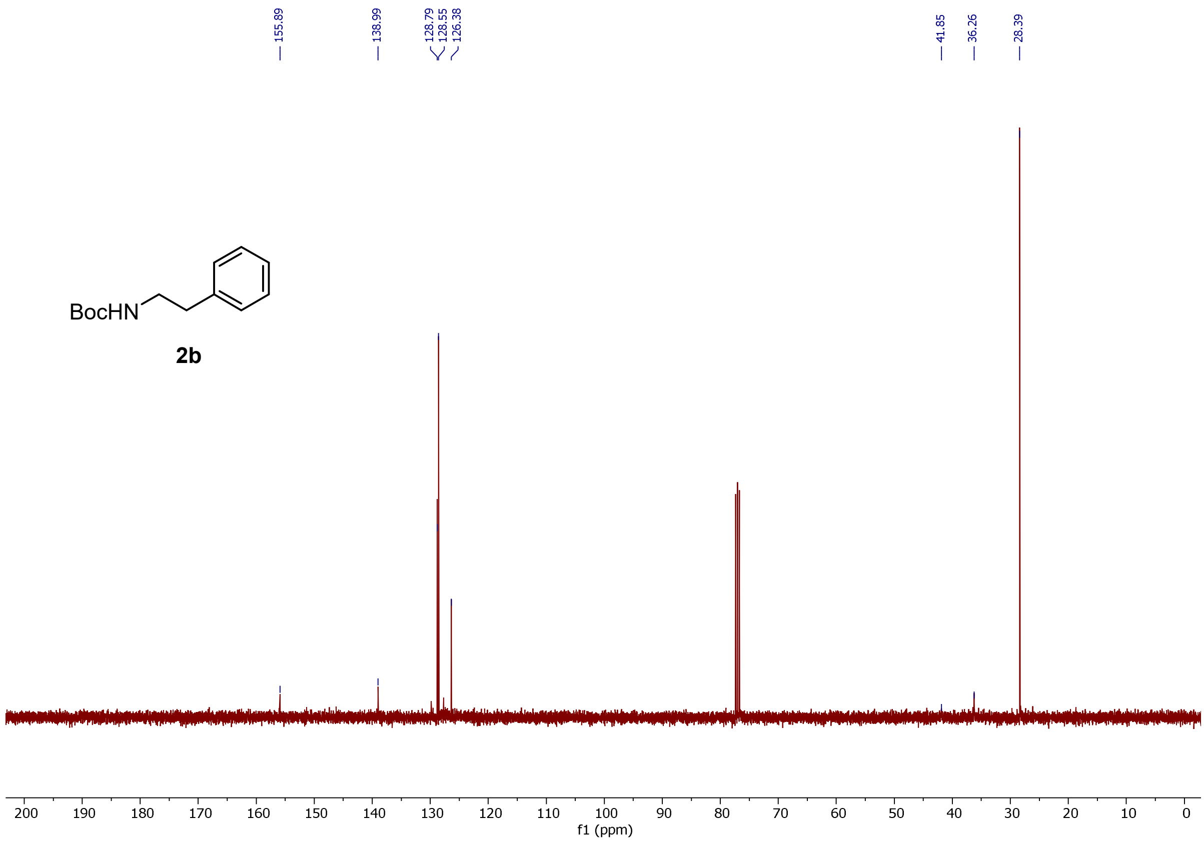

Figure S21. ^{13}C NMR (101 MHz, $CDCl_3$) of compound **2b**.

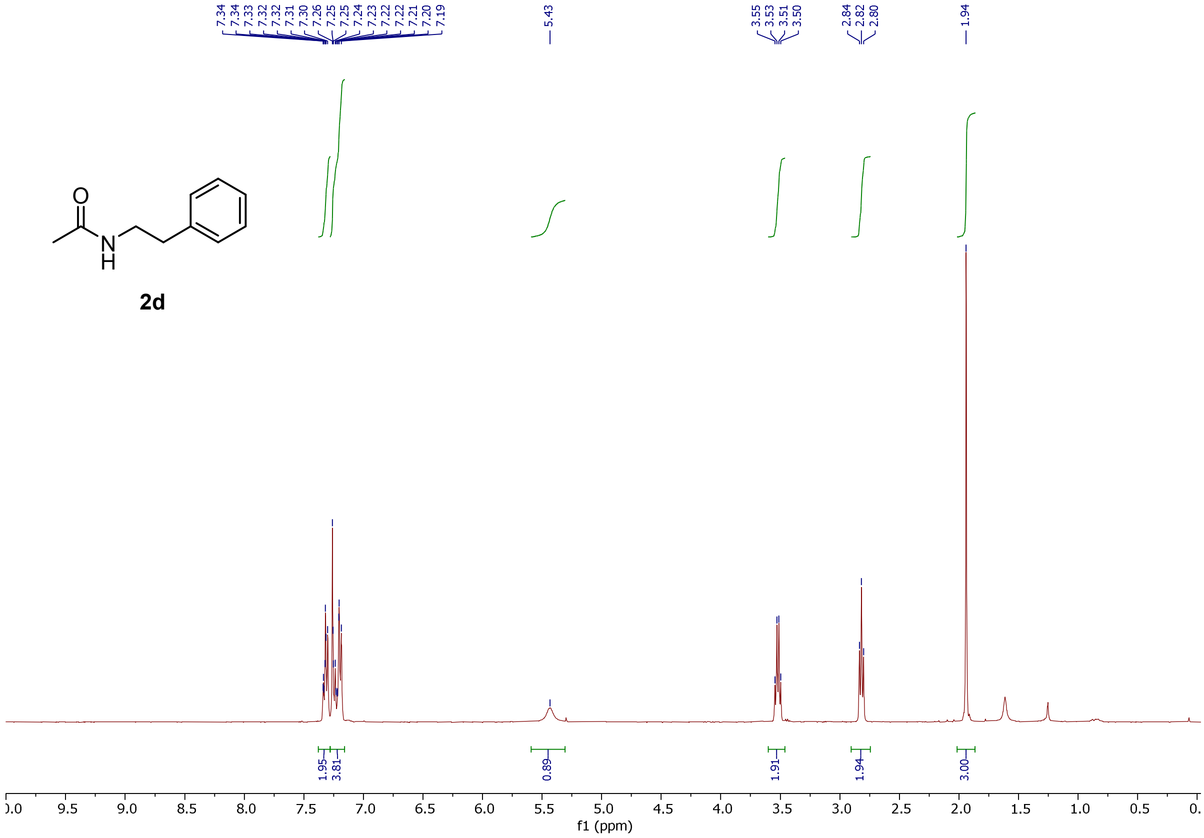

Figure S22. ^{1}H NMR (400 MHz, $CDCl_3$) of compound **2d**.

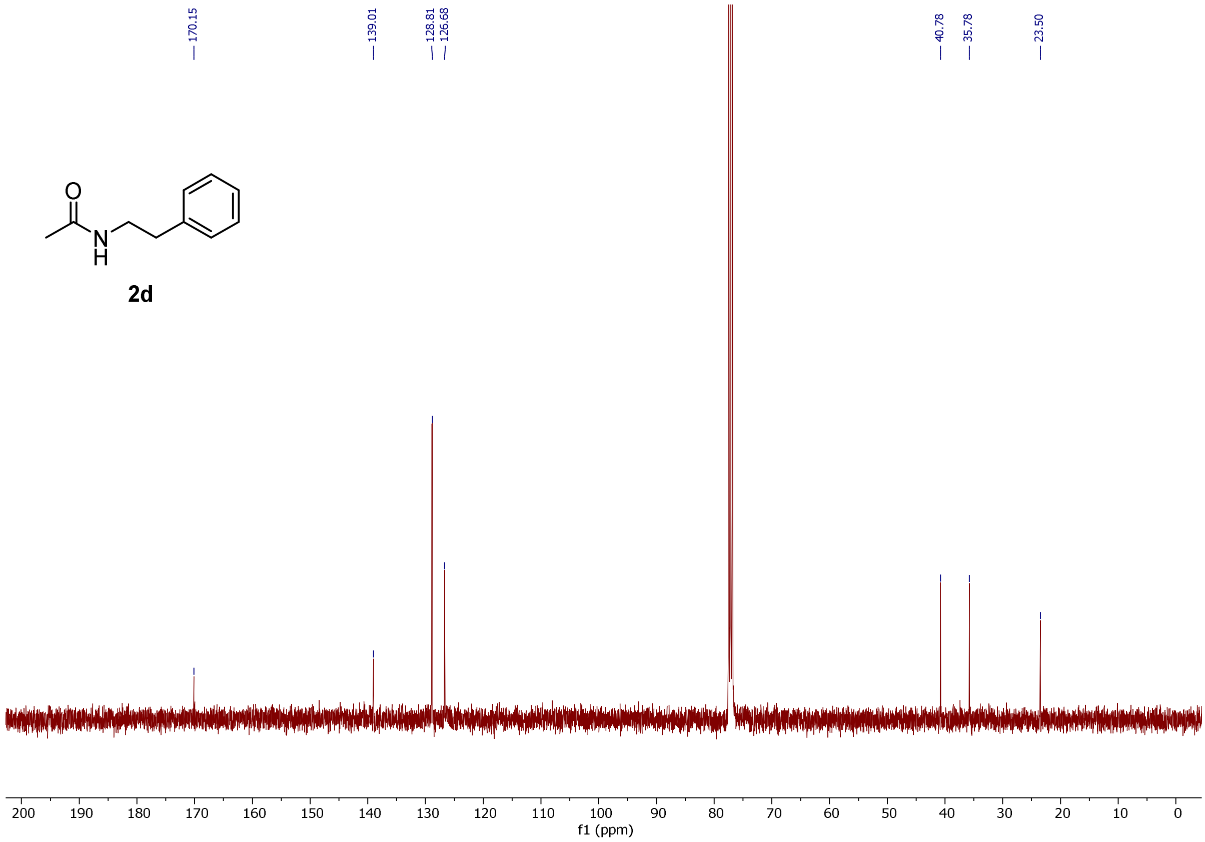

Figure S23. ^{13}C NMR (101 MHz, $CDCl_3$) of compound **2d**.

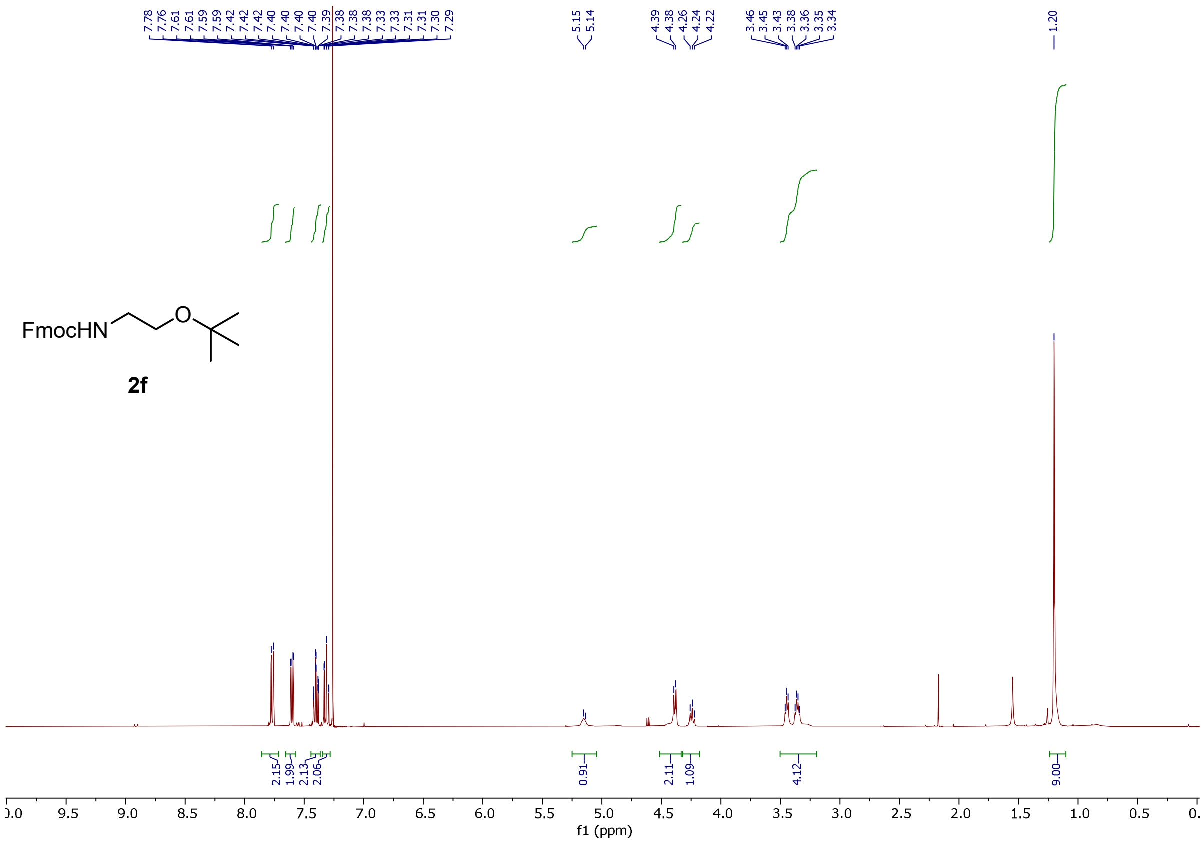

Figure S24. ^{1}H NMR (400 MHz, $CDCl_3$) of compound **2f**.

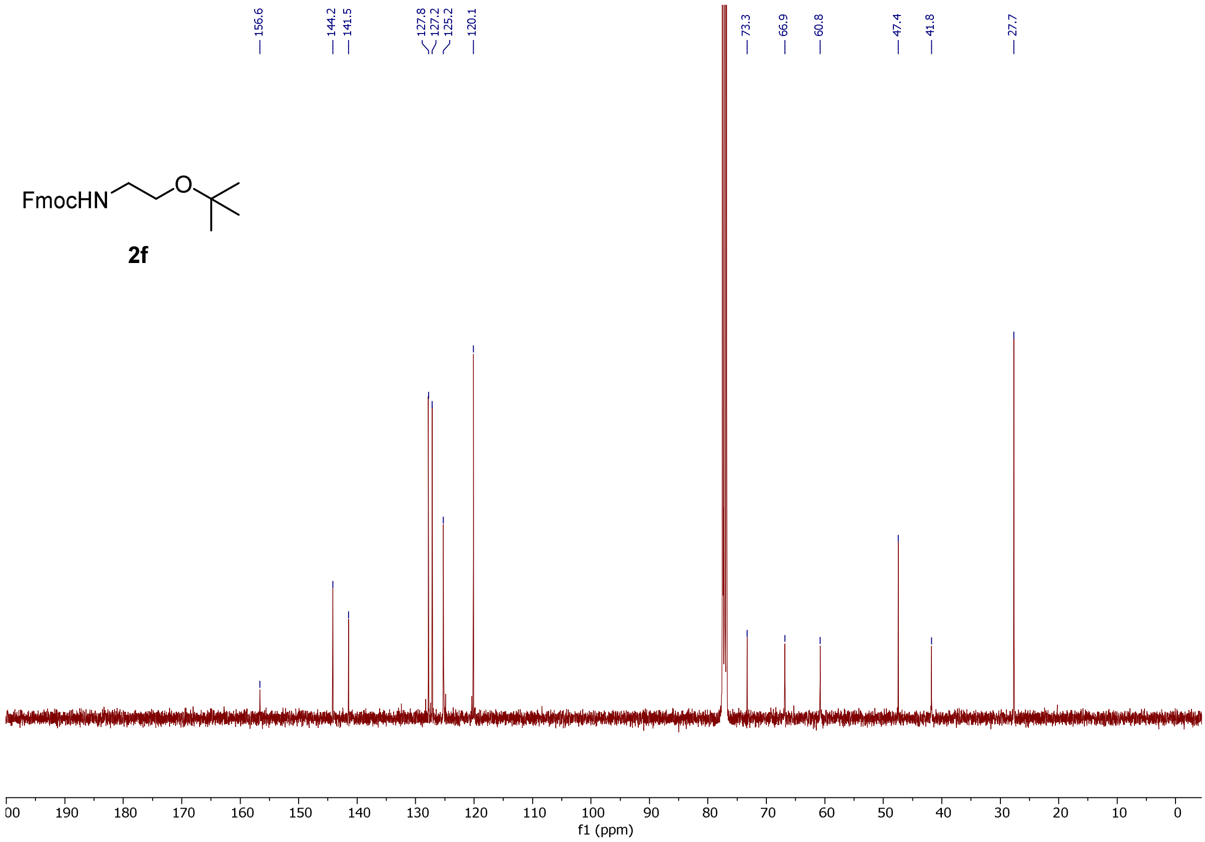

Figure S25. ^{13}C NMR (101 MHz, $CDCl_3$) of compound **2f**.

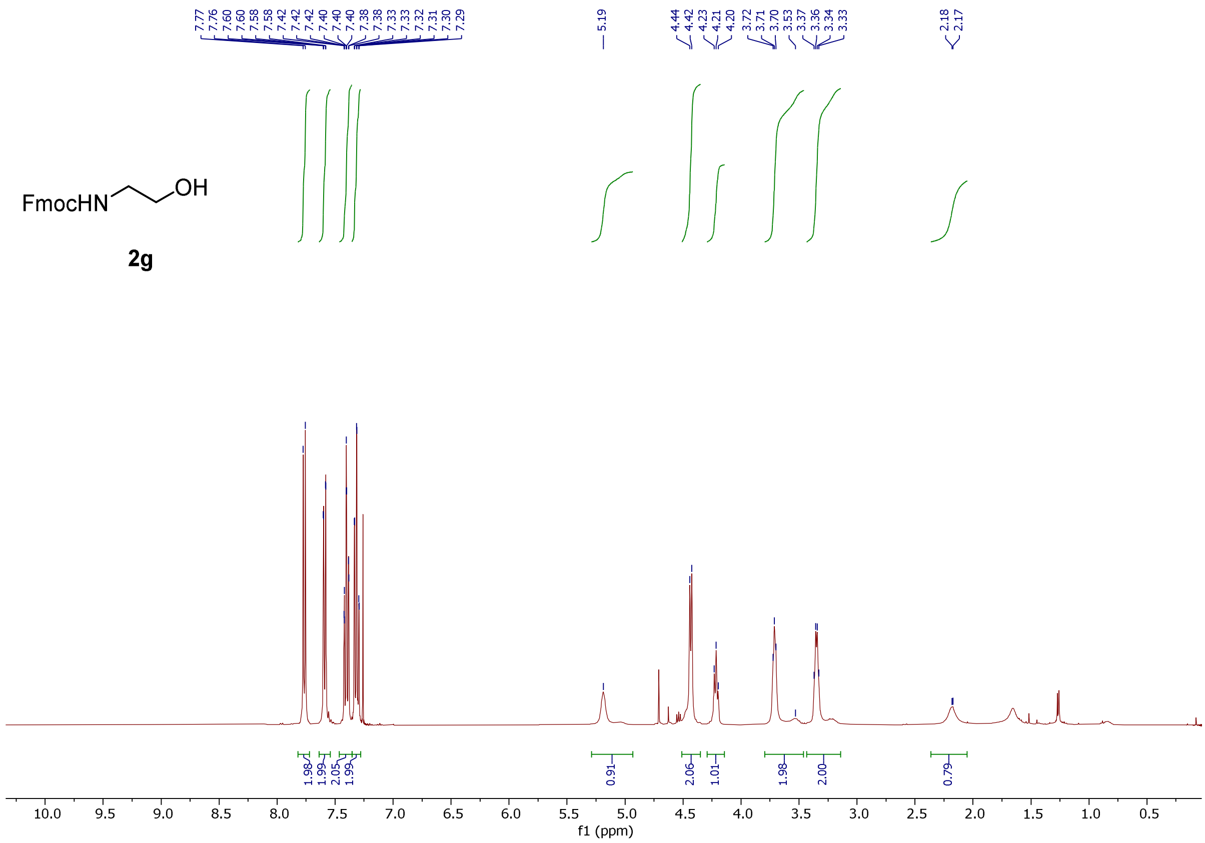

Figure S26. ^{1}H NMR (400 MHz, $CDCl_3$) of compound **2g**.

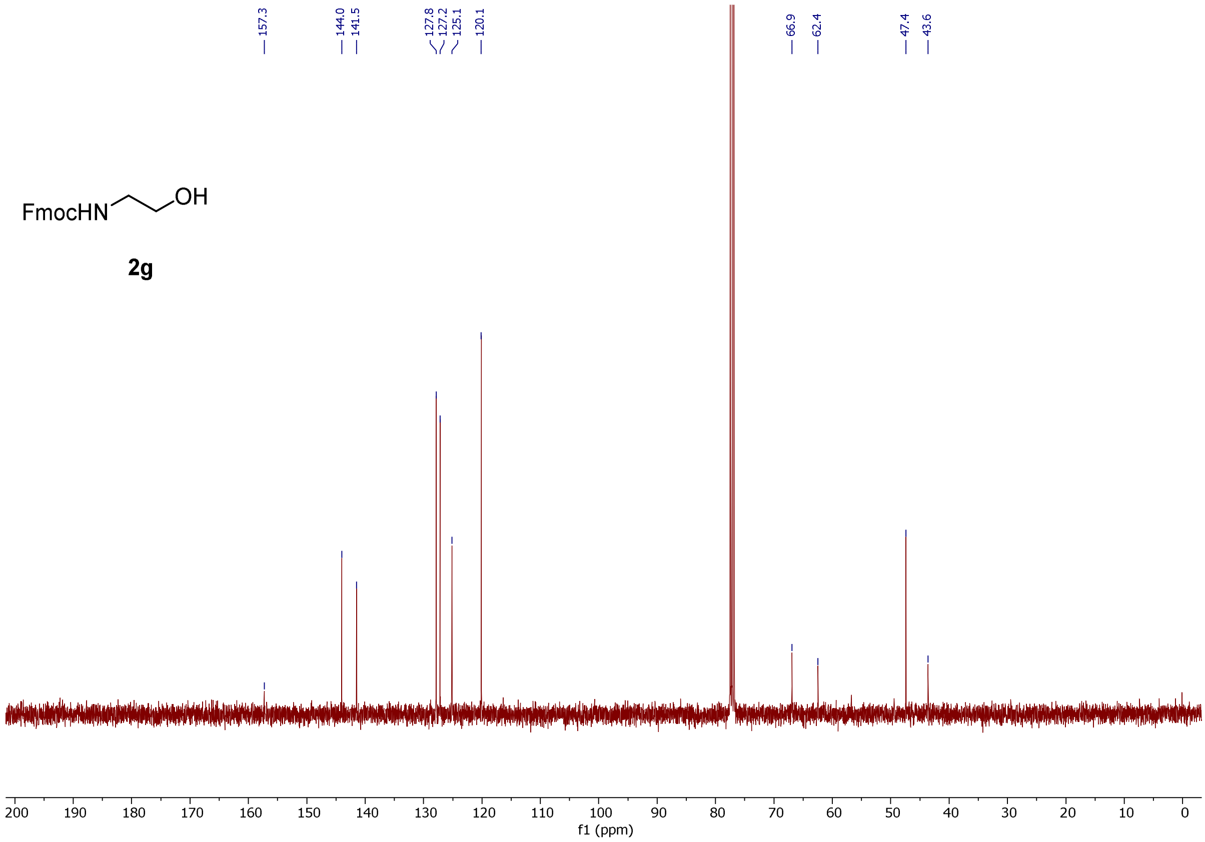

Figure S27. ^{13}C NMR (101 MHz, $CDCl_3$) of compound **2g**.

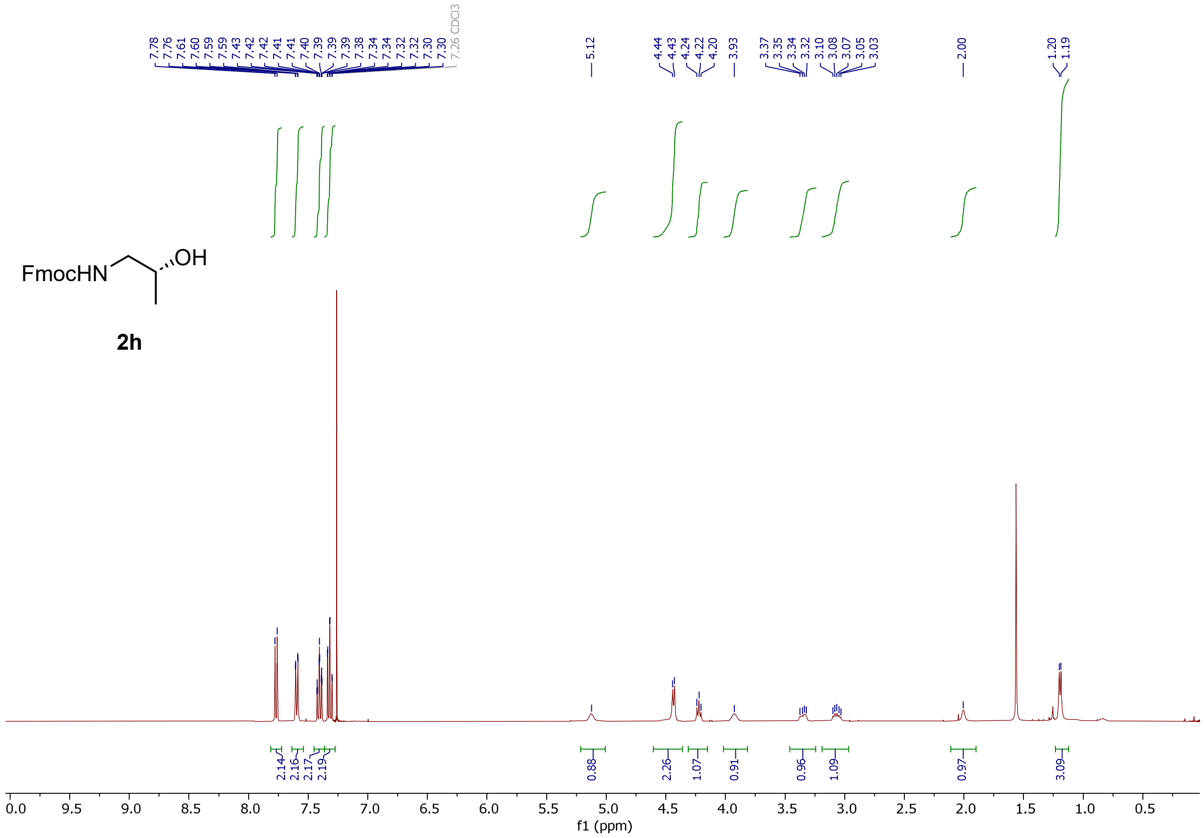

Figure S28. ^{1}H NMR (400 MHz, $CDCl_3$) of compound **2h**.

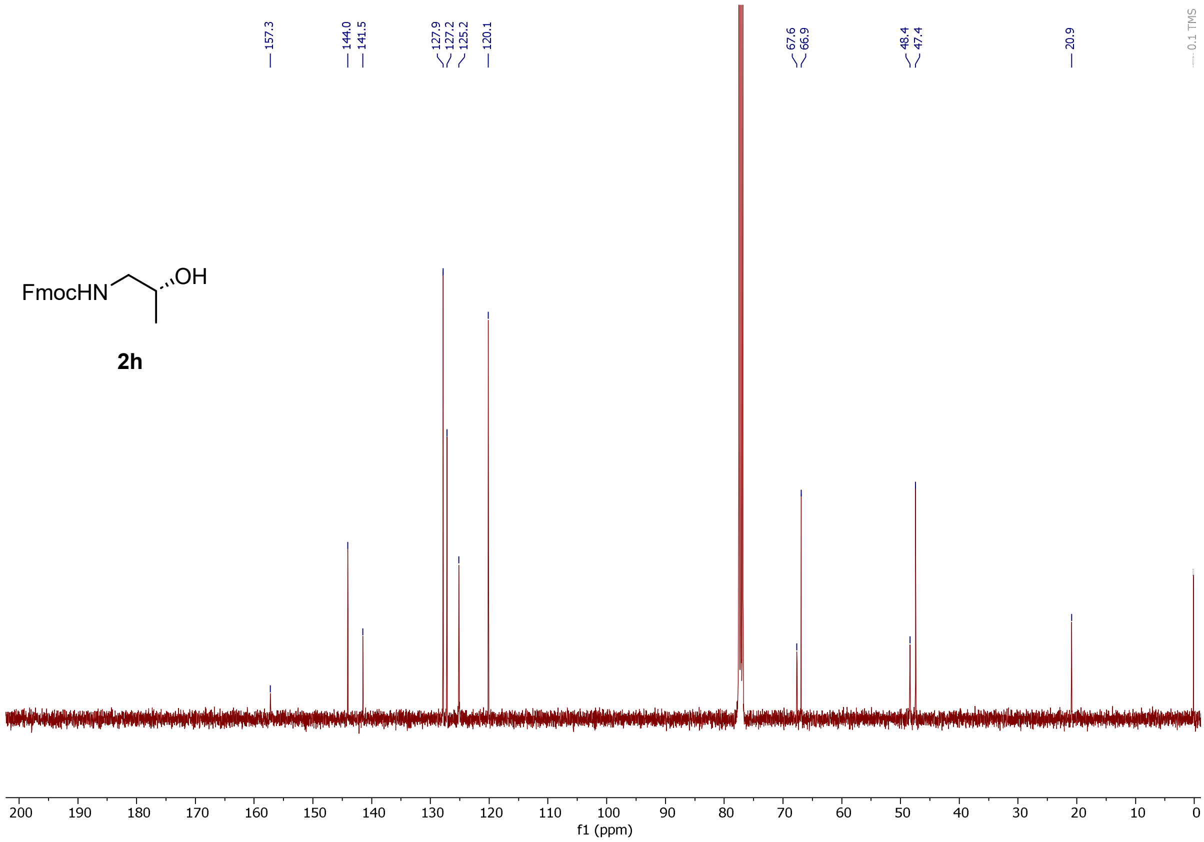

Figure S29. ^{13}C NMR (101 MHz, $CDCl_3$) of compound **2h**.

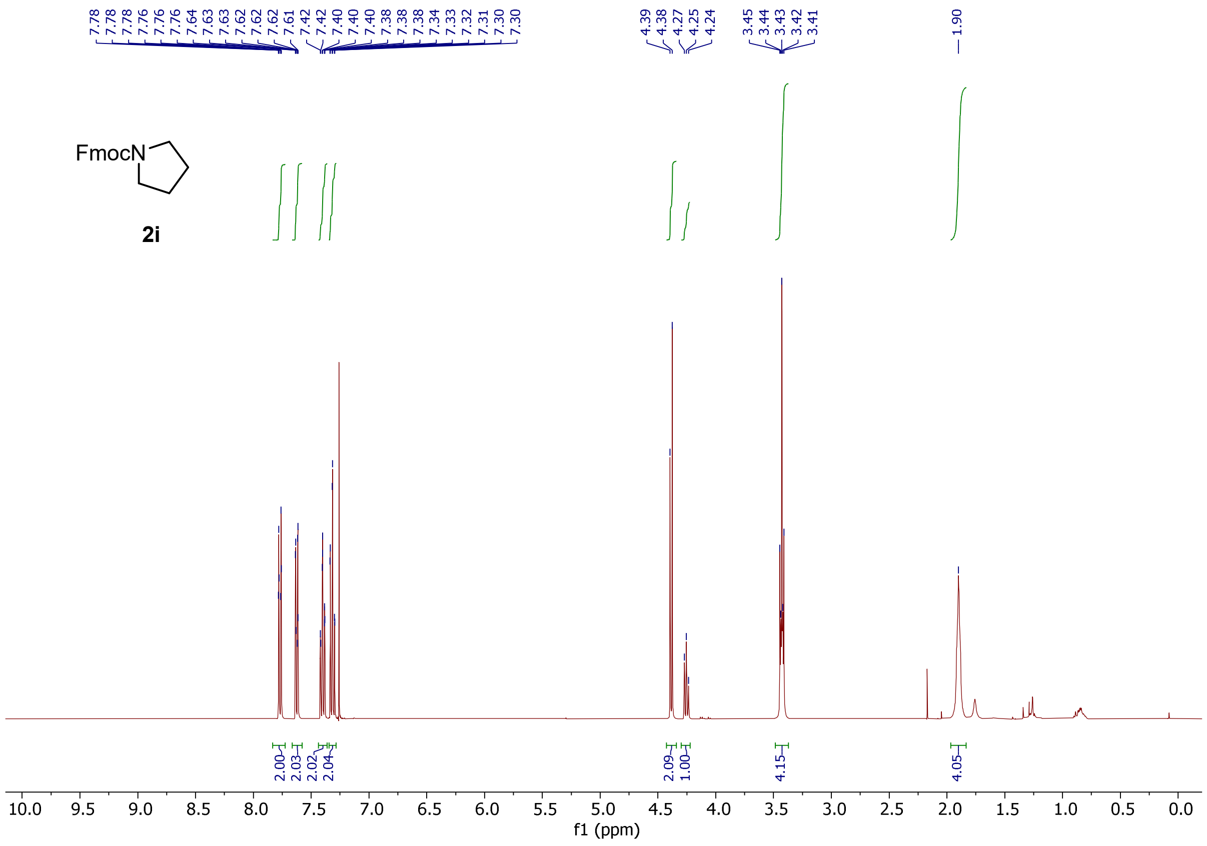

Figure S30. ^{1}H NMR (400 MHz, $CDCl_3$) of compound **2i**.

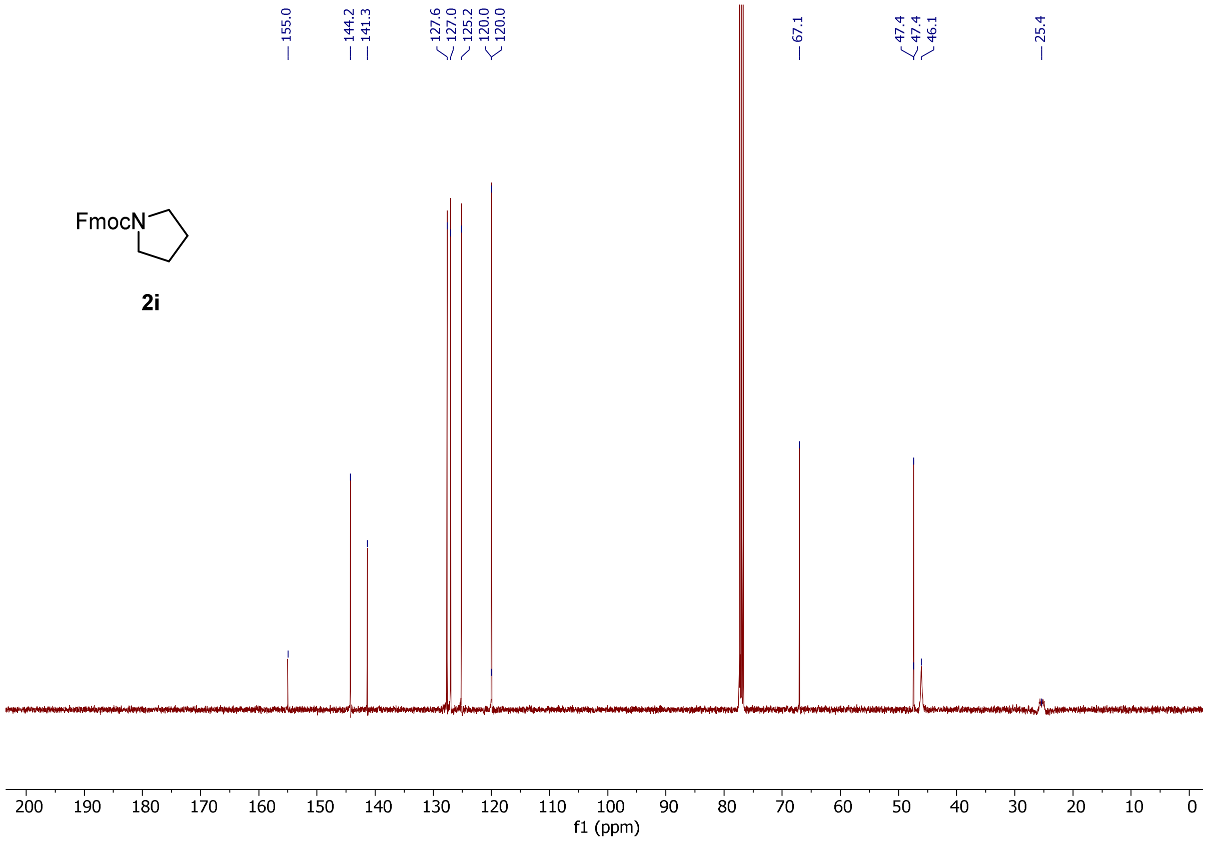

Figure S31. ^{13}C NMR (101 MHz, $CDCl_3$) of compound **2i**.

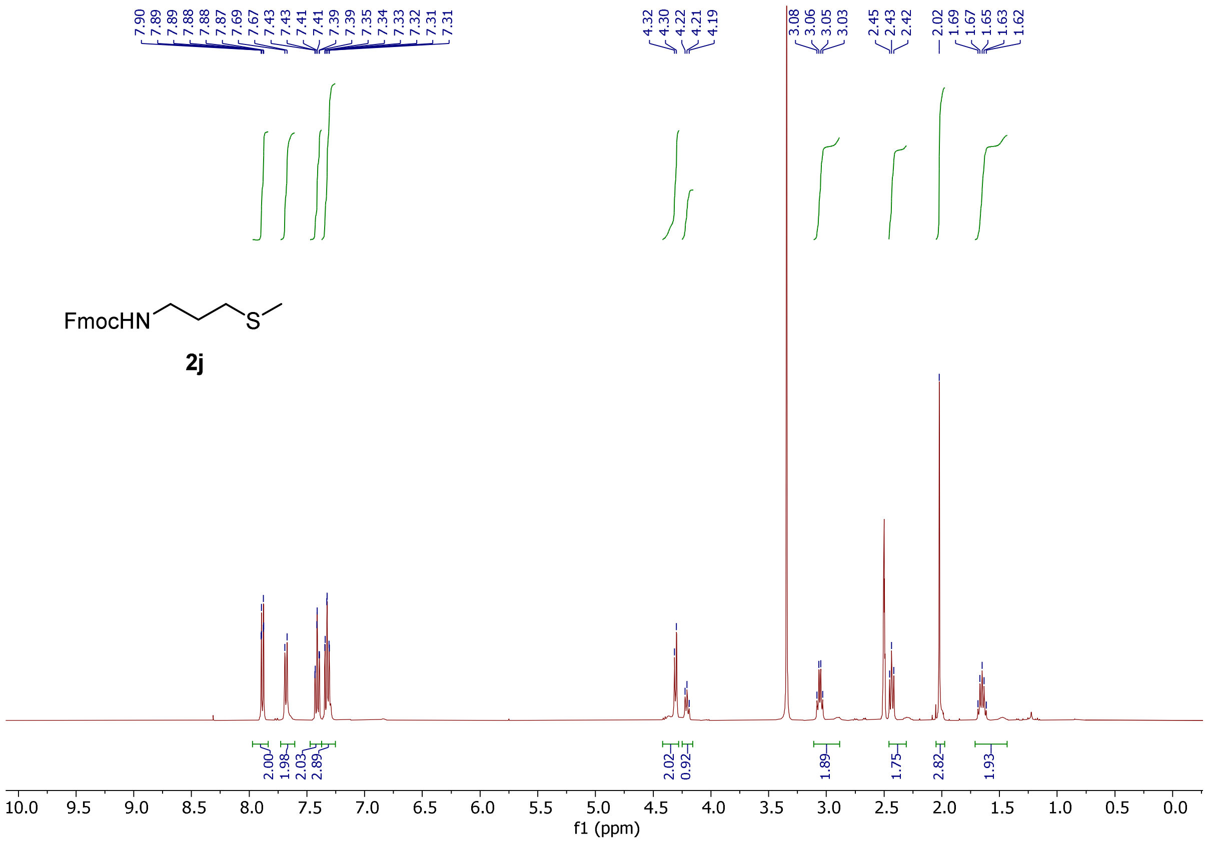

Figure S32. ^{1}H NMR (400 MHz, DMSO-d$_6$) of compound **2j**.

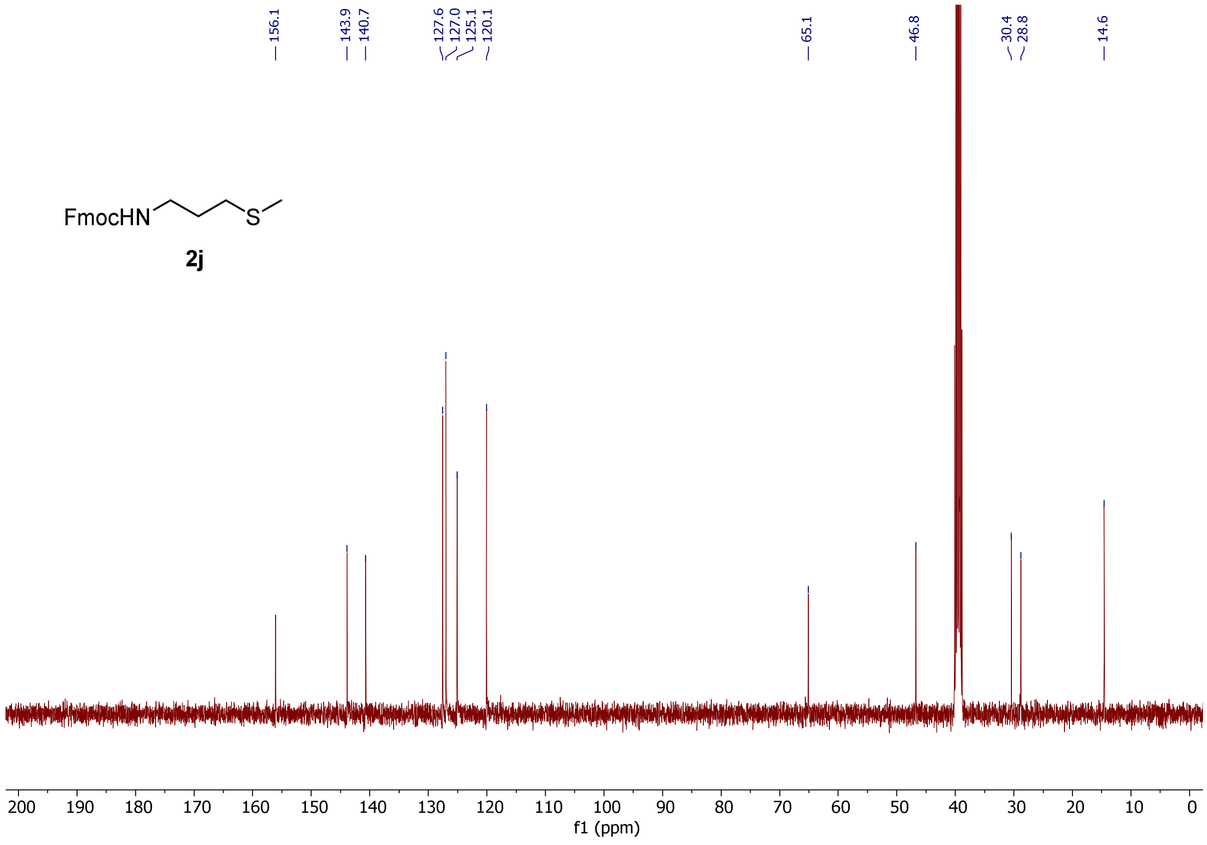

Figure S33. ^{13}C NMR (101 MHz, DMSO-d$_6$) of compound **2j**.

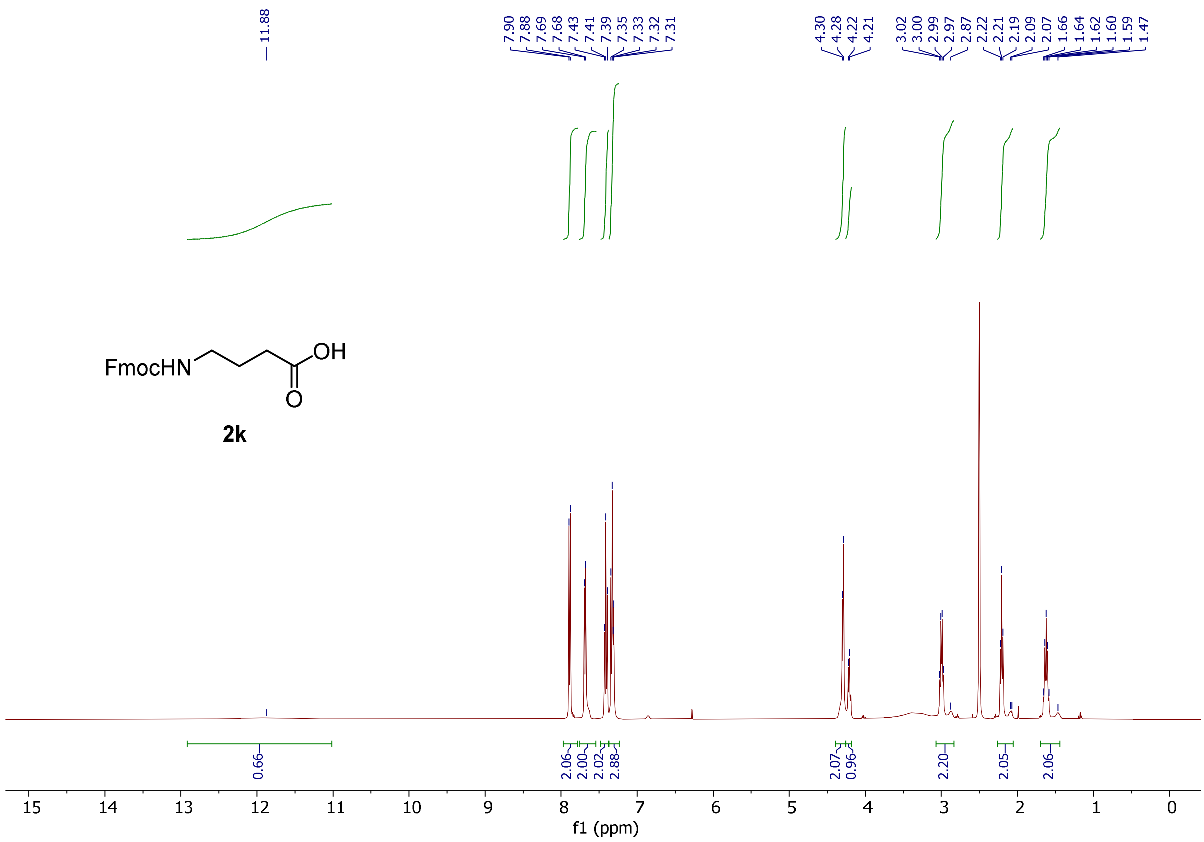

Figure S34. ^{1}H NMR (400 MHz, DMSO-d_6) of compound **2k**.

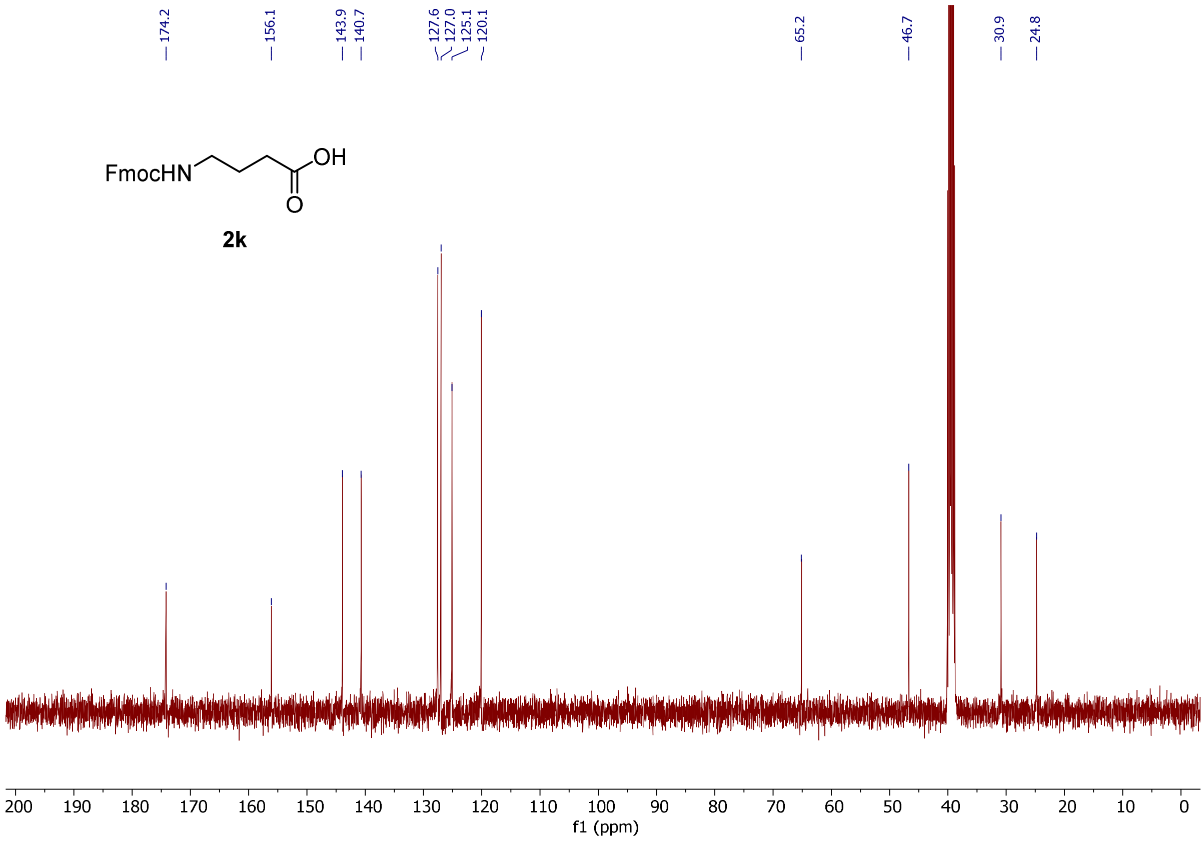

Figure S35. ^{13}C NMR (101 MHz, DMSO-d_6) of compound **2k**.

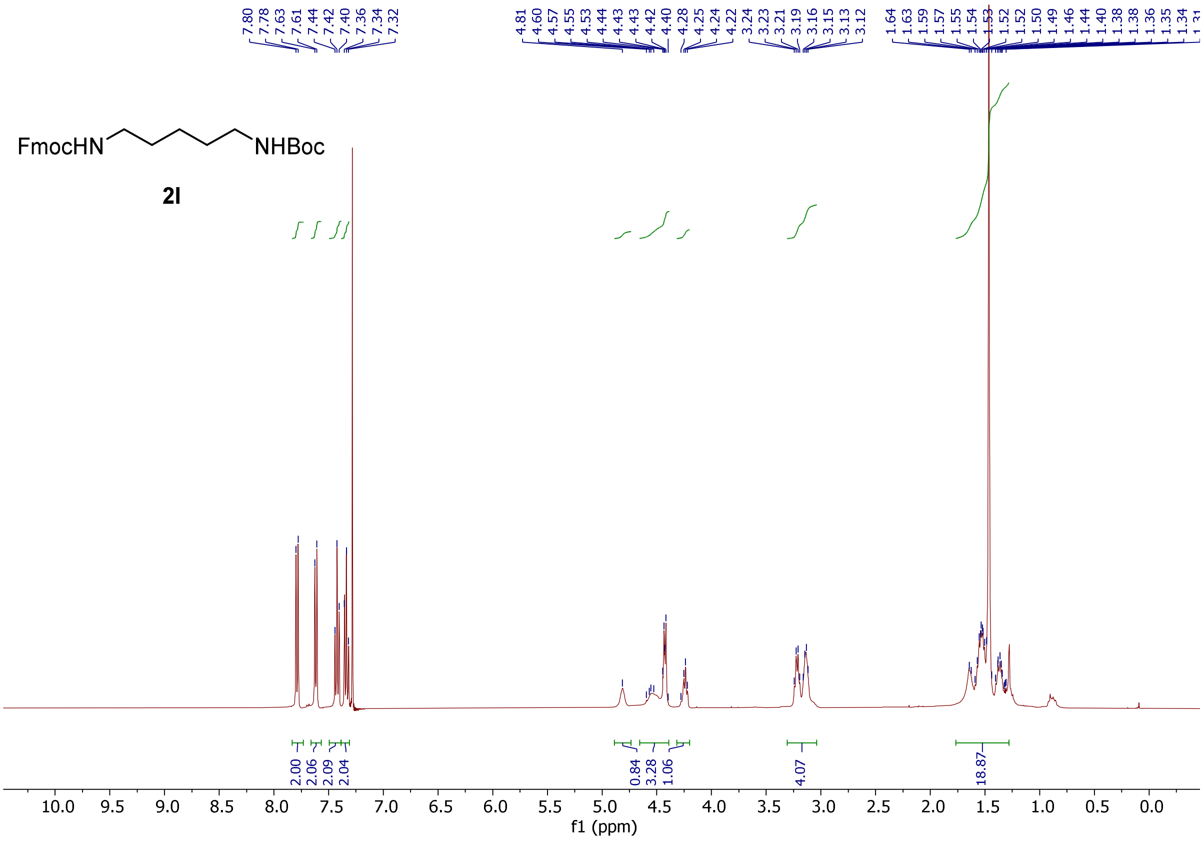

Figure S36. ^{1}H NMR (400 MHz, $CDCl_3$) of compound **2l**.

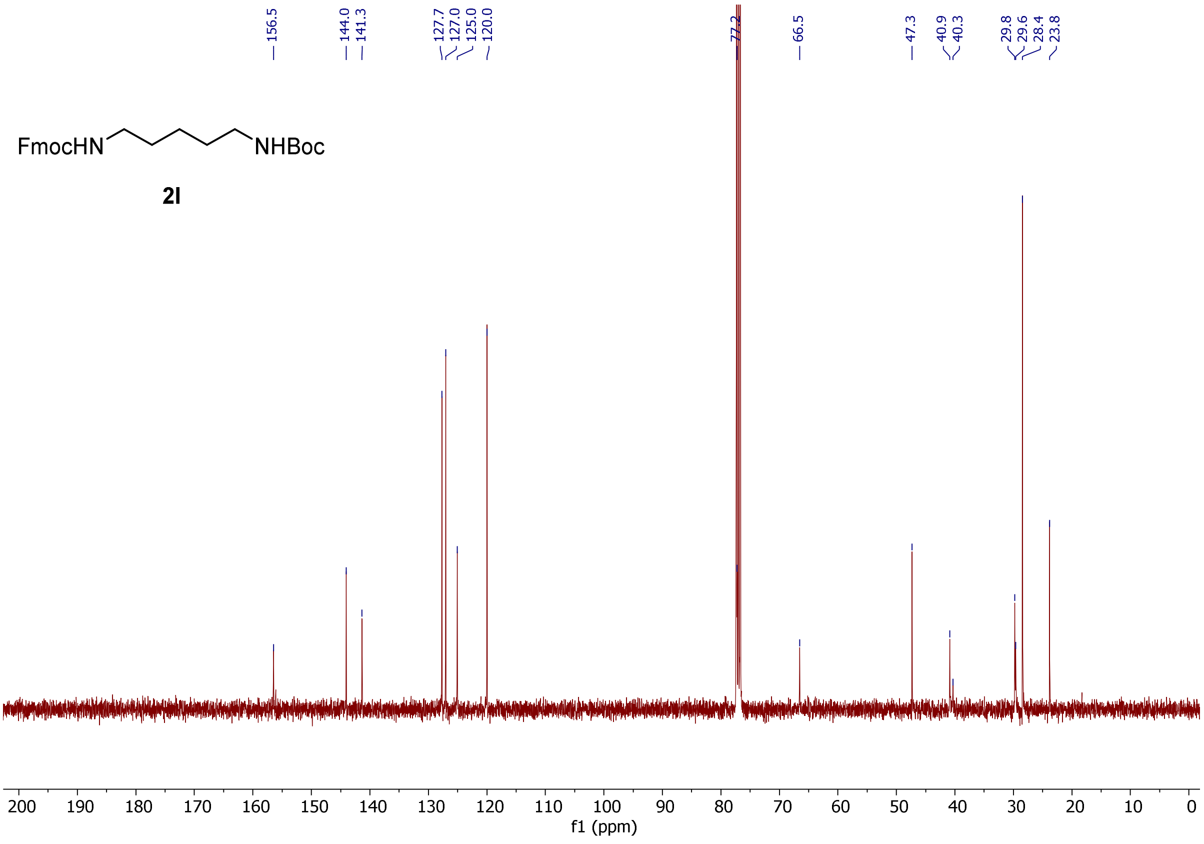

Figure S37. ^{13}C NMR (101 MHz, $CDCl_3$) of compound **2l**.

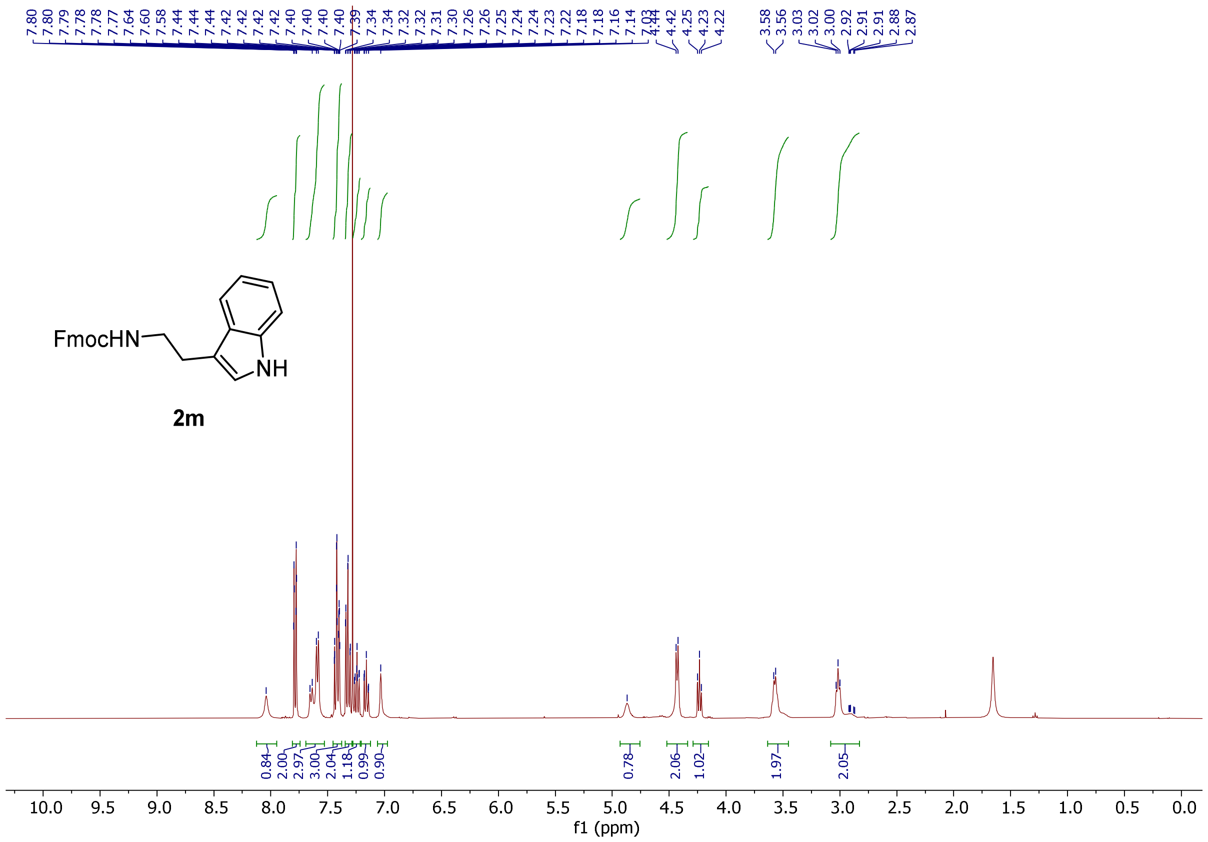

Figure S38. ^{1}H NMR (400 MHz, $CDCl_3$) of compound **2m**.

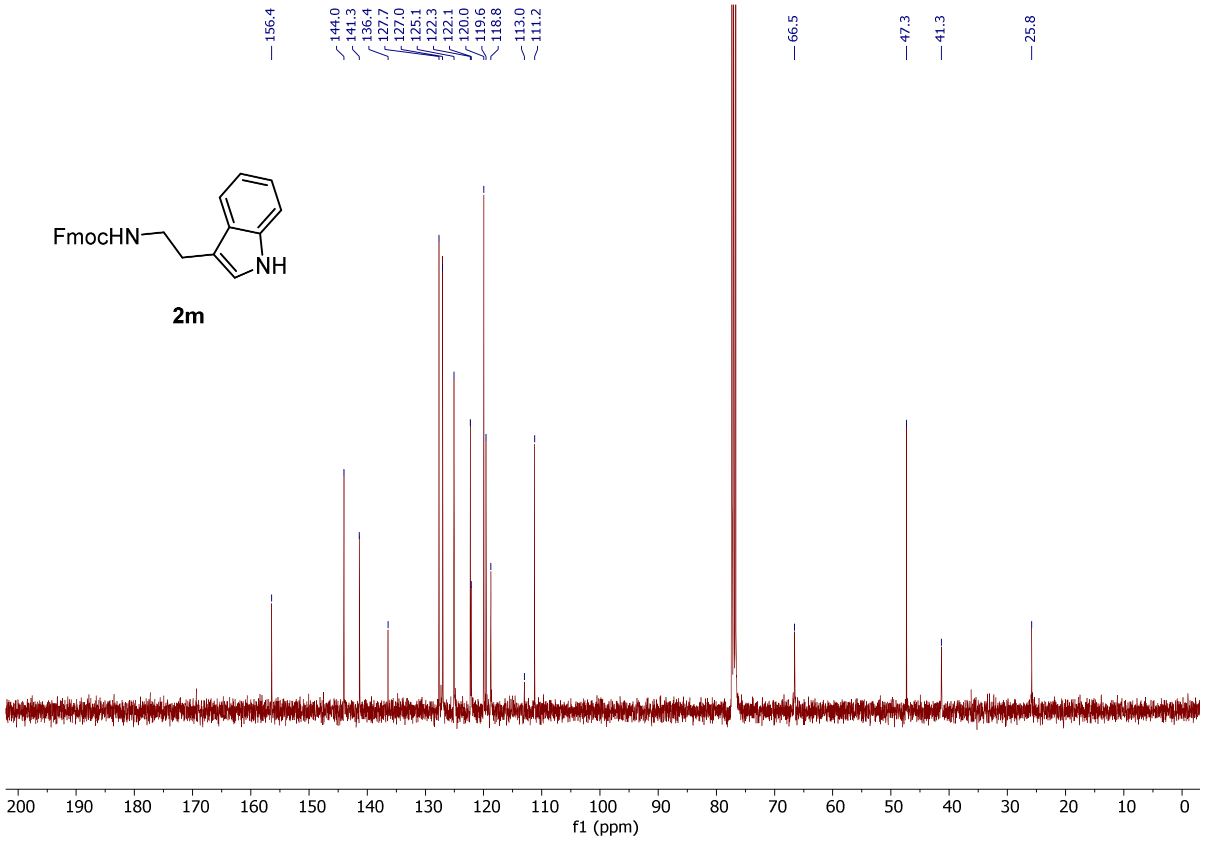

Figure S39. ^{13}C NMR (101 MHz, $CDCl_3$) of compound **2m**.
